# Cost-effectiveness of pembrolizumab plus weekly paclitaxel for PD-L1–positive platinum-resistant recurrent ovarian cancer in the United States and China

**DOI:** 10.3389/fphar.2026.1926275

**Published:** 2026-09-21

**Authors:** Feng Xuan, Haoze Bian, Minguang Huang, Yan Yang, Guodong Wang

**Affiliations:** 1 Department of Radiation Oncology, Zhuji Affiliated Hospital of Wenzhou Medical University, Shaoxing, China; 2 Department of Pharmacy, Zhuji Affiliated Hospital of Wenzhou Medical University, Shaoxing, China; 3 Department of Medical Oncology, Zhuji Affiliated Hospital of Wenzhou Medical University, Shaoxing, China

**Keywords:** cost-effectiveness analysis, incremental cost-effectiveness ratio, partitioned survival model, pembrolizumab, platinum-resistant ovarian cancer, quality-adjusted life-years

## Abstract

**Background:**

Pembrolizumab plus weekly paclitaxel, with or without bevacizumab, improves survival in programmed death ligand 1 (PD-L1)–positive platinum-resistant recurrent ovarian cancer, but its economic value remains uncertain. This study evaluated the cost-effectiveness of pembrolizumab plus weekly paclitaxel versus placebo plus weekly paclitaxel in patients with PD-L1 combined positive score ≥1 platinum-resistant recurrent ovarian cancer from the Chinese and United States (US) healthcare system perspectives.

**Methods:**

A partitioned survival model was developed using data from ENGOT-ov65/KEYNOTE-B96. Outcomes included costs, life-years (LYs), quality-adjusted life-years (QALYs), incremental cost-effectiveness ratios (ICERs), incremental net monetary benefits (INMBs), and incremental net health benefits (INHBs). Cost-effectiveness was assessed against willingness-to-pay (WTP) thresholds of $27,906/QALY in China and $150,000/QALY in the US. Deterministic and probabilistic sensitivity analyses, scenario analyses, and subgroup analyses were performed.

**Results:**

In China, pembrolizumab plus weekly paclitaxel increased costs from $31,241.22 to $127,168.48 and increased outcomes from 1.57 to 2.11 LYs and from 1.02 to 1.34 QALYs, yielding 0.54 additional LYs and 0.32 additional QALYs. The ICER was $295,671.88/QALY, with an INMB of −$86,873.48 and an INHB of −3.11 QALYs. In the US, costs increased from $199,488.96 to $451,893.63, and outcomes increased from 1.59 to 2.15 LYs and from 1.03 to 1.37 QALYs. The ICER was $746,255.61/QALY, with an INMB of −$201,670.44 and an INHB of −1.34 QALYs. Pembrolizumab cost and health-state utilities were the main ICER drivers. Probabilistic sensitivity analysis showed 0% cost-effectiveness probability at both WTP thresholds. Scenario and subgroup analyses confirmed the base-case findings. At current bevacizumab prices, pembrolizumab prices would need substantial reductions to meet country-specific WTP thresholds.

**Conclusion:**

Compared with placebo plus weekly paclitaxel, pembrolizumab plus weekly paclitaxel improved survival and QALYs in patients with PD-L1–positive platinum-resistant recurrent ovarian cancer but was unlikely to be cost-effective at current prices in either China or the US.

## 1 Introduction

Ovarian cancer imposes a considerable global burden, ranking third in incidence and second in mortality among gynecologic cancers, with 324,398 new cases and 206,839 deaths estimated in 2022 (Bray et al., 2024). China contributes 18.8% of worldwide cases and the United States (US) 5.6%, corresponding to the highest national burdens in Asia and the Americas, respectively (Kun et al., 2025). Despite progress in cytoreductive surgery, platinum chemotherapy, antiangiogenic therapy, and targeted maintenance strategies, most patients with advanced ovarian cancer experience recurrence, and platinum resistance develops in a substantial proportion (Eric et al., 2014; Shubin et al., 2026).

Platinum-resistant ovarian cancer is associated with poor responsiveness to later-line treatment and unfavorable survival. In this setting, conventional single-agent non-platinum chemotherapy provides limited benefit, with objective response rate (ORR) of approximately 10%–20%, median progression-free survival (PFS) of 3–4 months, and median overall survival (OS) near 12–13 months (A N et al., 2001; Shubin et al., 2026). Bevacizumab, an anti-VEGF antibody, could extend median OS to about 16.6 months in selected patients. However, this survival advantage is modest and requires careful consideration of toxicity (Eric et al., 2014). Immune checkpoint inhibitors (ICIs) have reshaped the treatment landscape of several solid tumors. Pembrolizumab added to chemotherapy significantly improved PFS and OS in patients with persistent, recurrent, or metastatic cervical cancer (Colombo et al., 2021). Similarly, in previously untreated advanced esophageal cancer, pembrolizumab plus chemotherapy provided sustained survival benefit over placebo plus chemotherapy in the intention-to-treat population, with median OS of 12.3 versus 9.8 months (hazard ratio [HR] 0.72 [95% CI 0.62–0.84]) and median PFS of 6.3 versus 5.8 months (HR 0.64 [95% CI 0.54–0.75]) (Shah et al., 2024). In recurrent ovarian cancer, ICI monotherapy has historically shown modest activity, with ORRs of approximately 8%–12% in recurrent disease (Andrea et al., 2018; U A et al., 2019).

Pembrolizumab is a humanized IgG4 monoclonal antibody against PD-1 that disrupts PD-1 binding to PD-L1 and PD-L2, enabling recovery of antitumor T-cell function (Kwok et al., 2016). Weekly paclitaxel may enhance antitumor immune responses by increasing tumor immunogenicity, promoting immunogenic cell death, and reducing immunosuppressive components within the tumor microenvironment, particularly when administered at lower, metronomic-like doses (Christina et al., 2016; Colombo et al., 2026; Somayeh et al., 2022). The phase 3 ENGOT-ov65/KEYNOTE-B96 trial provided the first pivotal evidence supporting this strategy in platinum-resistant recurrent ovarian cancer. In this multicentre, randomized, double-blind trial, pembrolizumab plus weekly paclitaxel, with or without bevacizumab, was compared with placebo plus weekly paclitaxel, with or without bevacizumab, in patients with platinum-resistant recurrent ovarian cancer who had received one or two prior systemic treatment regimens (Colombo et al., 2026). Among patients with PD-L1 combined positive score (CPS) of one or greater, pembrolizumab-based therapy improved PFS compared with placebo-based therapy, with a median PFS of 8.3 versus 7.2 months (HR 0.72 [95% CI 0.58–0.89]). OS was also improved, with a median OS of 18.2 versus 14.0 months (HR 0.76 [95% CI 0.61–0.94]) (Colombo et al., 2026; Colombo et al., 2025). In February 2026, the US Food and Drug Administration approved pembrolizumab in combination with paclitaxel, with or without bevacizumab, for adults with PD-L1 CPS ≥1 platinum-resistant epithelial ovarian, fallopian tube, or primary peritoneal carcinoma who had received one or two prior systemic regimens (FDA approves pembrolizumab with paclitaxel for platinum-resistant epithelial ovarian, fallopian tube, or primary peritoneal carcinoma, 2026). The European Commission subsequently approved the same regimen on 2 April 2026, for adults with PD-L1 CPS ≥1 platinum-resistant recurrent ovarian carcinoma after one or two previous systemic treatment regimens (European Commission, 2026). On 25 June 2026, the National Medical Products Administration of China also approved pembrolizumab in combination with paclitaxel, with or without bevacizumab, for adults with PD-L1 CPS ≥1 platinum-resistant epithelial ovarian, fallopian tube, or primary peritoneal carcinoma who had received one or two previous lines of systemic therapy. These approvals support the clinical relevance of evaluating the economic value of this regimen in both the US and China.

As more effective cancer therapies have emerged, the financial burden of oncology care has grown, particularly for regimens that require prolonged exposure to ICIs, cytotoxic chemotherapy, and possible bevacizumab. This issue is especially relevant for international decision-making, given substantial differences between the US and China in drug costs, reimbursement systems, healthcare utilization, willingness-to-pay (WTP) thresholds, and patients’ ability to pay. Therefore, in this study, we conducted an international cost-effectiveness analysis to evaluate pembrolizumab plus weekly paclitaxel for patients with PD-L1–positive platinum-resistant recurrent ovarian cancer from the perspectives of the US and Chinese healthcare systems. This analysis was designed to inform payer decision-making, clinical adoption, and pricing strategies for this newly approved immunotherapy-based regimen.

## 2 Materials and methods

### 2.1 Study design and analytical overview

We conducted an international cost-effectiveness analysis comparing pembrolizumab plus weekly paclitaxel with placebo plus weekly paclitaxel for platinum-resistant recurrent ovarian cancer from the healthcare perspectives of the US and China. The analysis was developed and reported in accordance with the Consolidated Health Economic Evaluation Reporting Standards (CHEERS) 2022 guideline (Supplementary Table S1).(Husereau et al., 2022) A partitioned survival model (PSM) with three mutually exclusive health states, including PFS, progressed disease (PD), and death, was constructed to estimate long-term costs and health outcomes (Figure 1A).

**FIGURE 1:**
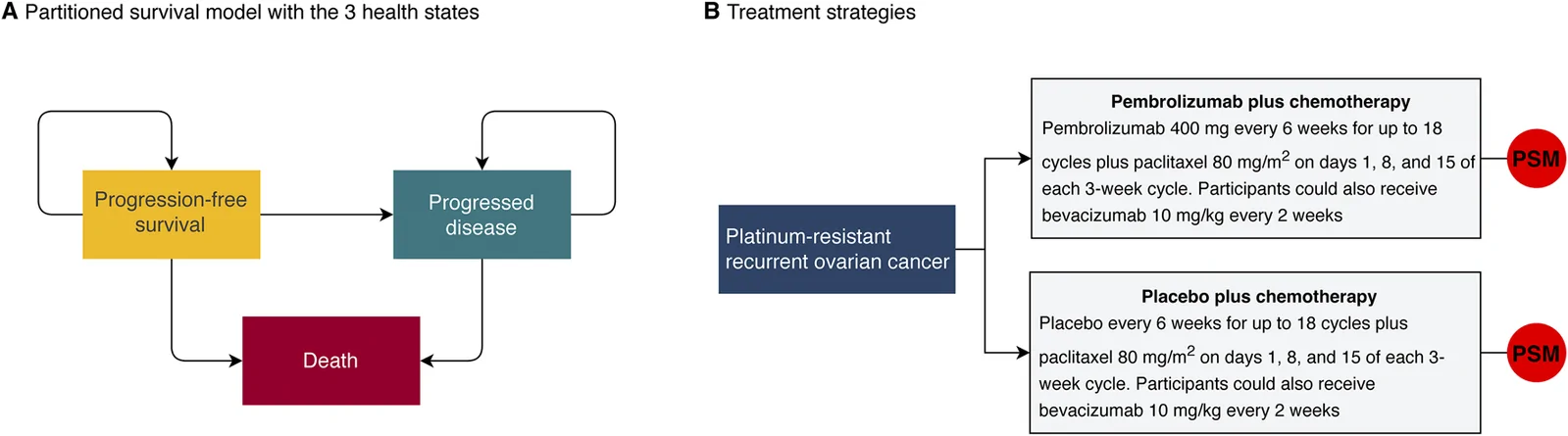
Partitioned survival model consisting of three health states. **(A)** Partitioned survival model overview. **(B)** Treatment strategies in ENGOT-ov65/KEYNOTE-B96 for PD-L1–positive platinum-resistant recurrent ovarian cancer.

All patient, treatment, efficacy, and safety inputs for the base-case analysis were derived from ENGOT-ov65/KEYNOTE-B96, a randomized, double-blind, phase 3 trial conducted at 187 gynecologic oncology centers across 25 countries in the Americas, Asia, Europe, and Oceania. Eligible patients were adults with histologically confirmed epithelial ovarian, fallopian tube, or primary peritoneal carcinoma who had received one or two prior systemic regimens, including at least one platinum-containing regimen, and who experienced disease progression within 6 months after the last platinum regimen. Between 13 December 2021, and 3 July 2023, 643 patients were randomly assigned to pembrolizumab plus paclitaxel (n = 322) or placebo plus paclitaxel (n = 321) in the intention-to-treat population (Figure 1B). 638 patients received at least one dose of study treatment and comprised the safety population. Data cutoff date was 5 September 2025, for the final analysis.

The base-case analysis used the PD-L1 CPS ≥1 population from ENGOT-ov65/KEYNOTE-B96. This population was selected because it represents the clinically relevant population included in the regulatory indications approved in both the US and China. Pembrolizumab in combination with paclitaxel, with or without bevacizumab, has been approved for adults with PD-L1 CPS ≥1 platinum-resistant epithelial ovarian, fallopian tube, or primary peritoneal carcinoma after one or two previous lines of systemic therapy. A scenario analysis subsequently examined the overall trial population enrolled in ENGOT-ov65/KEYNOTE-B96.

### 2.2 Model structure

A PSM was used because it directly links the proportions of patients in PFS, PD, and death to the modeled PFS and OS curves over time (Figure 1A). All patients entered the model in the PFS state. During each model cycle, the proportion of patients in the PFS state was estimated from the area under the PFS curve divided by the cycle length, whereas the proportion alive was estimated from the area under the OS curve divided by the cycle length. The proportion in the PD state was calculated as the difference between the areas under the OS and PFS curves divided by the cycle length, and the proportion in the death state was calculated as one minus the proportion alive.

The model adopted a lifetime horizon to capture all relevant downstream costs and consequences, and the time horizon was extended until more than 99% of the simulated cohort had died. A 42-day cycle length was used to align with the pembrolizumab dosing schedule of 400 mg administered intravenously every 6 weeks. Future costs and health outcomes were discounted annually at 3.0% for the US and 4.5% for China (Jing and Liu, 2025; Youwen et al., 2024). Costs were expressed in 2025 US dollars. Chinese costs were converted to US dollars using an exchange rate of 1 US dollar to 7.14 Chinese yuan.

### 2.3 Patients and treatment strategies

The intervention arm consisted of pembrolizumab, 400 mg intravenously every 6 weeks for up to 18 cycles, plus open-label weekly paclitaxel, 80 mg/m^2^ intravenously on days 1, 8, and 15 of each 21-day cycle (Figure 1B). The comparator arm consisted of placebo administered every 6 weeks for up to 18 cycles plus the same weekly paclitaxel regimen. Bevacizumab, 10 mg/kg intravenously every 2 weeks, was permitted at investigator discretion in both arms, consistent with the trial protocol. In the intention-to-treat population, bevacizumab was administered to 235 of 322 patients (73%) in the pembrolizumab plus paclitaxel arm and 236 of 321 patients (74%) in the placebo plus paclitaxel arm. Because arm-specific bevacizumab-use proportions were not separately reported for the PD-L1 CPS ≥1 population, these published intention-to-treat proportions were applied to the corresponding treatment arms in the base-case analysis. Bevacizumab acquisition and intravenous drug administration costs were weighted according to these arm-specific proportions. Thus, the intervention and comparator strategies were pembrolizumab plus weekly paclitaxel, with or without bevacizumab, and placebo plus weekly paclitaxel, with or without bevacizumab, respectively. Hereafter, these strategies are referred to as pembrolizumab plus chemotherapy and placebo plus chemotherapy, respectively. Treatment exposure, duration, and adverse event (AE) inputs were taken from ENGOT-ov65/KEYNOTE-B96. After disease progression, country-specific best supportive care costs were accrued during each cycle spent in the PD state. Terminal-care costs were applied separately as a one-time cost at death, based on the reduction in the overall-survival proportion between consecutive cycles. Although ENGOT-ov65/KEYNOTE-B96 collected information on subsequent anticancer treatment, the published article and supplementary materials did not provide sufficiently detailed, treatment-arm-specific data on subsequent regimens, treatment proportions, dosing, duration, or country-specific resource use. Therefore, specific subsequent anticancer therapies were not explicitly modelled, and best supportive care was used as a simplified representation of post-progression management.

### 2.4 Survival data reconstruction and extrapolation

Because patient-level survival data were unavailable, OS and PFS data for the base-case PD-L1 CPS ≥1 population were reconstructed from the final-analysis Kaplan–Meier curves presented in the Supplementary Appendix of ENGOT-ov65/KEYNOTE-B96. The final-analysis curves were selected because they provided the most mature available follow-up. The curves were digitized using WebPlotDigitizer (version 4.7) (WebPlotDigitizer, 2025), and reconstructed individual patient data were generated using established algorithms implemented in R software and the survival and survHE packages (Baio, 2020). Internal validation was performed by visually comparing the reconstructed Kaplan-Meier curves with the published trial curves (Supplementary Figure S1).

For each treatment group, seven standard parametric survival distributions (exponential, gamma, Gompertz, Weibull, generalized gamma, log-logistic, and log-normal) were fitted separately to the reconstructed OS and PFS data. Model selection was based on statistical goodness of fit, including the Akaike information criterion (AIC) and Bayesian information criterion (BIC), together with visual inspection of the fitted curves and the clinical plausibility of long-term extrapolation. AIC and BIC values for all candidate models are presented in Supplementary Table S2, and fitted survival curves are shown in Supplementary Figure S2; Supplementary Figure S3. The log-logistic distribution was ultimately chosen for OS and PFS in both treatment groups in the base-case analysis.

### 2.5 Costs and health utilities

Only direct medical costs were included. Cost categories comprised drug acquisition (ASP drug pricing files, 2025; Yaozhi Data, 2026; Paclitaxel Prices, Coupons, Copay Cards & Patient Assistance, 2025), intravenous drug administration (Kun et al., 2025; Shubin et al., 2026), laboratory testing (Shubin et al., 2026; Yijin et al., 2023), tumor imaging (Kun et al., 2025; Shubin et al., 2026), management of treatment-related AEs (Shubin et al., 2026; Yidong et al., 2025; Yijin et al., 2023; Yitian et al., 2025; Yuxuan et al., 2025), best supportive care (Kun et al., 2025; Shubin et al., 2026), and terminal care (Kun et al., 2025; Youwen et al., 2024). For the US, the acquisition costs of pembrolizumab and bevacizumab were obtained from the 2025 Average Sales Price drug pricing files published by the Centers for Medicare and Medicaid Services, accessed on 25 April 2026 (ASP drug pricing files, 2025). The price of paclitaxel was obtained from the Drugs.com price guide, accessed on 12 April 2026 (Paclitaxel Prices, Coupons, Copay Cards & Patient Assistance, 2025). For China, the prices of pembrolizumab, bevacizumab, and paclitaxel were obtained from the drug bid-winning price database of Yaozhi, accessed on 21 March 2026 (Yaozhi Data, 2026). When multiple bid-winning records or manufacturers were available, the median price was used after standardizing all prices to the same dose unit. For drugs dosed according to body weight or body surface area, country-specific anthropometric assumptions were applied. For the US, a body weight of 70 kg and a body surface area of 1.84 m^2^ were assumed (Youwen et al., 2023; Youwen et al., 2024). For China, a body weight of 61 kg and a body surface area of 1.64 m^2^ were used (Kun et al., 2025). These assumptions were then applied to calculate the corresponding drug doses and acquisition costs for all weight- or body-surface-area-based treatments.

Because trial-based health utility data were not reported in ENGOT-ov65/KEYNOTE-B96, utility weights for the PFS and PD states were derived from previously published economic evaluations (Shubin et al., 2026; Youwen et al., 2024). In the base-case analysis, utility values of 0.75 for PFS and 0.50 for PD were used (Shubin et al., 2026; Youwen et al., 2024). Death was assigned a utility of 0. AE-related disutility values were derived from published cost-effectiveness studies (Kun et al., 2025; Shubin et al., 2026; Yitian et al., 2025; Youwen et al., 2023; Youwen et al., 2024). Grade 1 and 2 AEs were assumed to have limited effects on long-term costs and health-related quality of life. Therefore, only grade 3 or higher AEs with an incidence of at least 5% in either treatment group were included in the model. AE management costs were applied as one-time costs during the first model cycle. AE-related QALY losses were calculated by multiplying the treatment-specific incidence of each AE by its corresponding disutility and duration expressed in years, and the resulting duration-adjusted QALY decrements were applied as one-time adjustments during the first model cycle. Because ENGOT-ov65/KEYNOTE-B96 did not report event-specific AE durations, these parameters were obtained from published economic studies (Rui et al., 2022; Zhao et al., 2023). In the absence of suitable published estimates, the durations of peripheral neuropathy and fatigue were assumed to be 21 days, corresponding to one paclitaxel treatment cycle. The baseline values, uncertainty ranges, probability distributions, and sources or assumptions for all AE-duration parameters are provided in Supplementary Table S3. All other base-case values, uncertainty ranges, probability distributions, valuation methods, and corresponding sources are presented in Table 1.

**TABLE 1:** Summary of model inputs and ranges.

Parameters	Baseline value (range)		Distribution	Source
	The United States	China		
Clinical parameters of model in the PD-L1 CPS of 1 or higher population				
Log-logistic OS survival model of pembrolizumab-chemotherapy group	Shape = 1.93056, Scale = 17.99017		Fixed	(Colombo et al., 2026)
Log-logistic PFS survival model of pembrolizumab-chemotherapy group	Shape = 1.93708, Scale = 8.61780		Fixed	(Colombo et al., 2026)
Log-logistic OS survival model of placebo-chemotherapy group	Shape = 2.02677, Scale = 14.48729		Fixed	(Colombo et al., 2026)
Log-logistic PFS survival model of placebo-chemotherapy group	Shape = 2.08014, Scale = 6.97026		Fixed	(Colombo et al., 2026)
Risk of grade 3–5 AEs in pembrolizumab plus chemotherapy				
Anemia	0.12 (0.10–0.14)		Beta	(Colombo et al., 2026)
Peripheral neuropathy	0.05 (0.04–0.06)		Beta	(Colombo et al., 2026)
Fatigue	0.05 (0.04–0.06)		Beta	(Colombo et al., 2026)
White blood cell count decreased	0.12 (0.10–0.14)		Beta	(Colombo et al., 2026)
Neutropenia	0.04 (0.04–0.05)		Beta	(Colombo et al., 2026)
Neutrophil count decreased	0.18 (0.15–0.22)		Beta	(Colombo et al., 2026)
Risk of grade 3–5 AEs in chemotherapy				
Anemia	0.08 (0.06–0.09)		Beta	(Colombo et al., 2026)
Peripheral neuropathy	0.06 (0.05–0.07)		Beta	(Colombo et al., 2026)
Fatigue	0.06 (0.05–0.07)		Beta	(Colombo et al., 2026)
White blood cell count decreased	0.09 (0.07–0.11)		Beta	(Colombo et al., 2026)
Neutropenia	0.14 (0.11–0.17)		Beta	(Colombo et al., 2026)
Neutrophil count decreased	0.14 (0.11–0.16)		Beta	(Colombo et al., 2026)
Estimates of utility and disutility				
Utility of PFS	0.75 (0.60–0.90)		Beta	(Shubin et al., 2026; Youwen et al., 2024)
Utility of PD	0.50 (0.40–0.60)		Beta	(Shubin et al., 2026; Youwen et al., 2024)
Disutility of anemia	0.073 (0.0584–0.0876)		Beta	(Youwen et al., 2024)
Disutility of peripheral neuropathy	0.185 (0.139–0.231)		Beta	(Yitian et al., 2025)
Disutility of fatigue	0.070 (0.056–0.084)		Beta	(Shubin et al., 2026)
Disutility of white blood cell count decreased	0.090 (0.072–0.108)		Beta	(Youwen et al., 2023)
Disutility of neutropenia	0.090 (0.072–0.108)		Beta	(Kun et al., 2025; Shubin et al., 2026)
Disutility of neutrophil count decreased	0.090 (0.072–0.108)		Beta	(Kun et al., 2025; Shubin et al., 2026)
Drug cost ($)				
Pembrolizumab	60.291 (48.2328–72.3492) per 1 mg	2,507.87 (2,006.29 to 3,009.44) per 100 mg	Gamma	(ASP drug pricing files, 2025; Yaozhi Data, 2026)
Bevacizumab	73.201 (58.5608–87.8412) per 10 mg	139.50 (111.60–167.39) per 100 mg	Gamma	(ASP drug pricing files, 2025; Yaozhi Data, 2026)
Paclitaxel	9.81 (7.848–11.772) per 30 mg	5.68 (4.54–6.81) per 30 mg	Gamma	(Yaozhi Data, 2026; Paclitaxel Prices, Coupons, Copay Cards & Patient Assistance, 2025)
AE management cost per event ($)				
Anemia	4,953.00 (3,962.40 to 5,943.60)	465.09 (372.07–558.11)	Gamma	(Yijin et al., 2023; Yuxuan et al., 2025)
Peripheral neuropathy	136.87 (109.50–164.24)	58.19 (46.55–69.83)	Gamma	(Yidong et al., 2025; Yitian et al., 2025)
Fatigue	313.45 (250.76–376.14)	15.90 (12.72–19.08)	Gamma	(Shubin et al., 2026; Yijin et al., 2023)
Neutrophil count decreased	5,321.00 (4,256.80 to 6,385.20)	138.77 (111.02–166.53)	Gamma	(Yijin et al., 2023; Yuxuan et al., 2025)
White blood cell count decreased	5,321.00 (4,256.80 to 6,385.20)	113.79 (91.03–136.55)	Gamma	(Yijin et al., 2023; Yuxuan et al., 2025)
Neutropenia	5,321.00 (4,256.80 to 6,385.20)	138.77 (111.02–166.53)	Gamma	(Yijin et al., 2023; Yuxuan et al., 2025)
Cost of the laboratory test per cycle ($)	450.00 (360.00–540.00)	166.85 (133.48–200.22)	Gamma	(Yijin et al., 2023)
Cost of Tumor imaging per cycle ($)	525.00 (420.00–630.00)	98.00 (78.40–117.60)	Gamma	(Kun et al., 2025; Shubin et al., 2026)
Cost of intravenous drug administration per cycle ($)	345.00 (276.00–414.00)	65.00 (52.00–78.00)	Gamma	(Kun et al., 2025; Shubin et al., 2026)
Cost of best supportive care per cycle ($)	6535.50 (5228.40–7842.60)	1,429.00 (1,143.20 to 1,714.80)	Gamma	(Kun et al., 2025; Shubin et al., 2026)
Cost of terminal care per patient ($)	56,210.00 (44,968.00 to 67,452.00)	1,773.00 (1,418.40 to 2,127.60)	Gamma	(Kun et al., 2025; Youwen et al., 2024)
Other parameters				
Body surface area (m^2^)	1.84 (1.472–2.208)	1.64 (1.312–1.968)	Normal	(Kun et al., 2025; Youwen et al., 2024)
Weight, kg	70 (35–105)	61 (50–73)	Normal	(Kun et al., 2025; Youwen et al., 2023)
Discount rate (%)	3.0 (0–5.0)	4.5 (0–5.0)	Fixed	(Jing and Liu, 2025; Youwen et al., 2024)

Per-cycle costs were applied once during each 42-day model cycle. The intravenous drug administration cost represented only the cost associated with administering anticancer drugs and did not include laboratory testing, tumor imaging, follow-up, supportive care, or other disease-management costs. Abbreviation: CPS, combined positive score; OS, overall survival; PFS, progression-free survival; AE, adverse event; PD, progressed disease.

### 2.6 Cost-effectiveness outcomes

The primary outcomes were total costs, life-years (LYs), quality-adjusted life-years (QALYs), incremental cost-effectiveness ratios (ICERs), incremental net health benefit (INHB), and incremental net monetary benefit (INMB) for pembrolizumab plus chemotherapy compared with placebo plus chemotherapy. ICERs were calculated as the incremental cost divided by the corresponding incremental LYs or QALYs. INHB and INMB were calculated as: $INHB\left(\lambda \right)=\Delta E-\frac{\Delta C}{\lambda }$ , $INMB\left(\lambda \right)=\Delta E\times \lambda -\Delta C$ , where $\Delta C=\mu C_{1}-\mu C_{0}$ and $\Delta E=\mu E_{1}-\mu E_{0}$ . *μCi* and *μEi* denote total costs and QALYs for treatment strategy *i*, respectively; *i* = 1 indicates pembrolizumab plus chemotherapy and *i* = 0 indicates placebo plus chemotherapy; and λ denotes the WTP threshold. WTP thresholds were set at $150,000 per QALY for the US and $27,906 per QALY for China (Jing and Liu, 2025; Shubin et al., 2026). The Chinese threshold represented twice the 2025 *per capita* gross domestic product (GDP) of $13,953, consistent with the recommendation of the China Guidelines for Pharmacoeconomic Evaluations (2025) (Jing and Liu, 2025). The 2025 *per capita* GDP was obtained from the National Bureau of Statistics of China (National Data: Annual Data, 2026).

### 2.7 Sensitivity analyses

To examine the robustness of the base-case results, one-way and two-way deterministic sensitivity analyses and a probabilistic sensitivity analysis were performed. In one-way sensitivity analyses, key model parameters were varied across prespecified ranges, defined as ±20% of the base-case values when empirical uncertainty estimates were unavailable. Results were summarized using tornado diagrams.

A two-way deterministic analysis was conducted to evaluate the joint effects of pembrolizumab and bevacizumab acquisition prices on the cost-effectiveness results. The prices of both drugs were varied simultaneously from zero to their respective base-case values, while all other model parameters were held constant. For each price combination, the ICER for pembrolizumab plus weekly paclitaxel versus placebo plus weekly paclitaxel was compared with the country-specific WTP threshold. The results were presented as two-dimensional price-threshold plots for China and the US.

Probabilistic sensitivity analysis was performed with 1,000 Monte Carlo simulations, in which all parameters were sampled simultaneously from prespecified probability distributions. Beta distributions were assigned to utilities and AE probabilities, and gamma distributions were assigned to cost parameters. Country-specific discount rates were held fixed. The results of the probabilistic sensitivity analysis were presented as incremental cost-effectiveness scatterplots and cost-effectiveness acceptability curves.

### 2.8 Scenario analyses

Two scenario analyses were conducted to evaluate structural and population-related uncertainty. In scenario 1, the base-case log-logistic survival functions were replaced with the second-best-fitting parametric distributions identified during model selection. In scenario 2, the overall ENGOT-ov65/KEYNOTE-B96 population was evaluated instead of the PD-L1 CPS ≥1 population used in the base-case analysis.

### 2.9 Subgroup analyses

Subgroup analyses were performed by incorporating subgroup-specific HRs for OS and PFS for pembrolizumab plus chemotherapy vs. placebo plus chemotherapy. Prespecified factors included age, Eastern Cooperative Oncology Group (ECOG) performance status, poly ADP-ribose polymerase (PARP) inhibitor exposure, PD-L1 CPS subgroup, bevacizumab use, and platinum-free interval. Because subgroup-specific survival curves for the comparator arm were unavailable, the baseline comparator survival estimated in the overall model was retained, and subgroup-specific HRs were applied to adjust survival for the pembrolizumab arm.

All data processing, model development, and figure generation were conducted using R (version 4.4.1), and Microsoft Excel.

## 3 Results

### 3.1 Base-case analysis

The base-case results are shown in Table 2. In China, pembrolizumab plus chemotherapy increased total costs from $31,241.22 to $127,168.48, with an incremental cost of $95,927.26. It also increased survival from 1.57 to 2.11 LYs and from 1.02 to 1.34 QALYs, corresponding to gains of 0.54 LYs and 0.32 QALYs. The resulting ICER was $295,671.88/QALY, which exceeded the China-specific WTP threshold of $27,906/QALY. The INMB was −$86,873.48, and the INHB was −3.11 QALYs. In the US, pembrolizumab plus chemotherapy increased total costs from $199,488.96 to $451,893.63, yielding an incremental cost of $252,404.67. The regimen provided 2.15 LYs and 1.37 QALYs, compared with 1.59 LYs and 1.03 QALYs for placebo plus chemotherapy, resulting in gains of 0.56 LYs and 0.34 QALYs. The ICER was $746,255.61/QALY, exceeding the US WTP threshold of $150,000/QALY. The INMB was −$201,670.44, and the INHB was −1.34 QALYs.

**TABLE 2:** Summary of cost and outcome results in the base-case analysis and the scenario analysis.

Strategies	Cost ($)	Overall LYs	Overall QALYs	ICER ($/LY)	ICER($/QALY)	INMB ($)	INHB (QALYs)
Base-case results							
China							
Placebo plus chemotherapy	31,241.22	1.57	1.02	Reference	Reference	Reference	Reference
Pembrolizumab plus chemotherapy	127,168.48	2.11	1.34	178,730.43	295,671.88	−86,873.48	−3.11
The United States							
Placebo plus chemotherapy	199,488.96	1.59	1.03	Reference	Reference	Reference	Reference
Pembrolizumab plus chemotherapy	451,893.63	2.15	1.37	449,529.82	746,255.61	−201,670.44	−1.34
Scenario 1: the second-best-fitting parametric distributions							
China							
Placebo plus chemotherapy	31,249.20	1.57	1.02	Reference	Reference	Reference	Reference
Pembrolizumab plus chemotherapy	127,168.27	2.11	1.34	178,773.71	295,769.00	−86,869.04	−3.11
The United States							
Placebo plus chemotherapy	199,524.73	1.59	1.03	Reference	Reference	Reference	Reference
Pembrolizumab plus chemotherapy	451,897.51	2.15	1.37	449,601.45	746,444.87	−201,657.82	−1.34
Scenario 2: in the overall population							
China							
Placebo plus chemotherapy	31,101.59	1.57	1.01	Reference	Reference	Reference	Reference
Pembrolizumab plus chemotherapy	123,865.12	2.01	1.29	211,424.99	343,013.13	−85,216.70	−3.05
The United States	​	​	​	​	​	​	​
Placebo plus chemotherapy	198,745.55	1.59	1.02	Reference	Reference	Reference	Reference
Pembrolizumab plus chemotherapy	440,023.69	2.05	1.31	524,929.95	855,796.16	−198,988.03	−1.33

Abbreviation: LY, life-year; QALY, quality-adjusted life year; ICER, incremental cost-effectiveness ratio; INMB, incremental net monetary benefits; INHB, incremental net health benefits.

### 3.2 Sensitivity analyses

#### 3.2.1 One-way sensitivity analysis

One-way sensitivity analyses identified the cost of pembrolizumab, utility of PFS, and utility of PD as the main drivers of the ICER in both countries (Figure 2). In China, varying the cost of pembrolizumab per 100 mg produced the widest ICER range, from $242,005.97/QALY to $349,337.79/QALY (Figure 2A). The next most influential parameters were the utility of PFS, which changed the ICER from $267,758.01/QALY to $330,083.17/QALY, and the utility of PD, which changed the ICER from $269,791.16/QALY to $327,044.87/QALY. The discount rate had a smaller effect, with ICERs ranging from $265,844.57/QALY to $298,999.71/QALY. Other parameters, including the cost of bevacizumab, best supportive care, body weight, adverse-event risks, and monitoring costs, had limited effects on the model results. Across all tested parameter ranges, the ICER remained above the China-specific WTP threshold of $27,906/QALY. In the US, the cost of pembrolizumab per 1 mg was also the most influential parameter, with ICERs ranging from $622,054.46/QALY to $870,456.76/QALY (Figure 2B). The utilities of PFS and PD were the next largest contributors, generating ICER ranges of $676,881.21/QALY to $831,474.46/QALY and $679,847.72/QALY to $827,041.46/QALY, respectively. The discount rate changed the ICER from $700,754.08/QALY to $776,790.26/QALY. In contrast, costs related to bevacizumab, best supportive care, tumor imaging, laboratory testing, intravenous drug administration, paclitaxel, and AEs showed comparatively modest effects. In all scenarios, the ICER remained higher than the US WTP threshold of $150,000/QALY.

**FIGURE 2:**
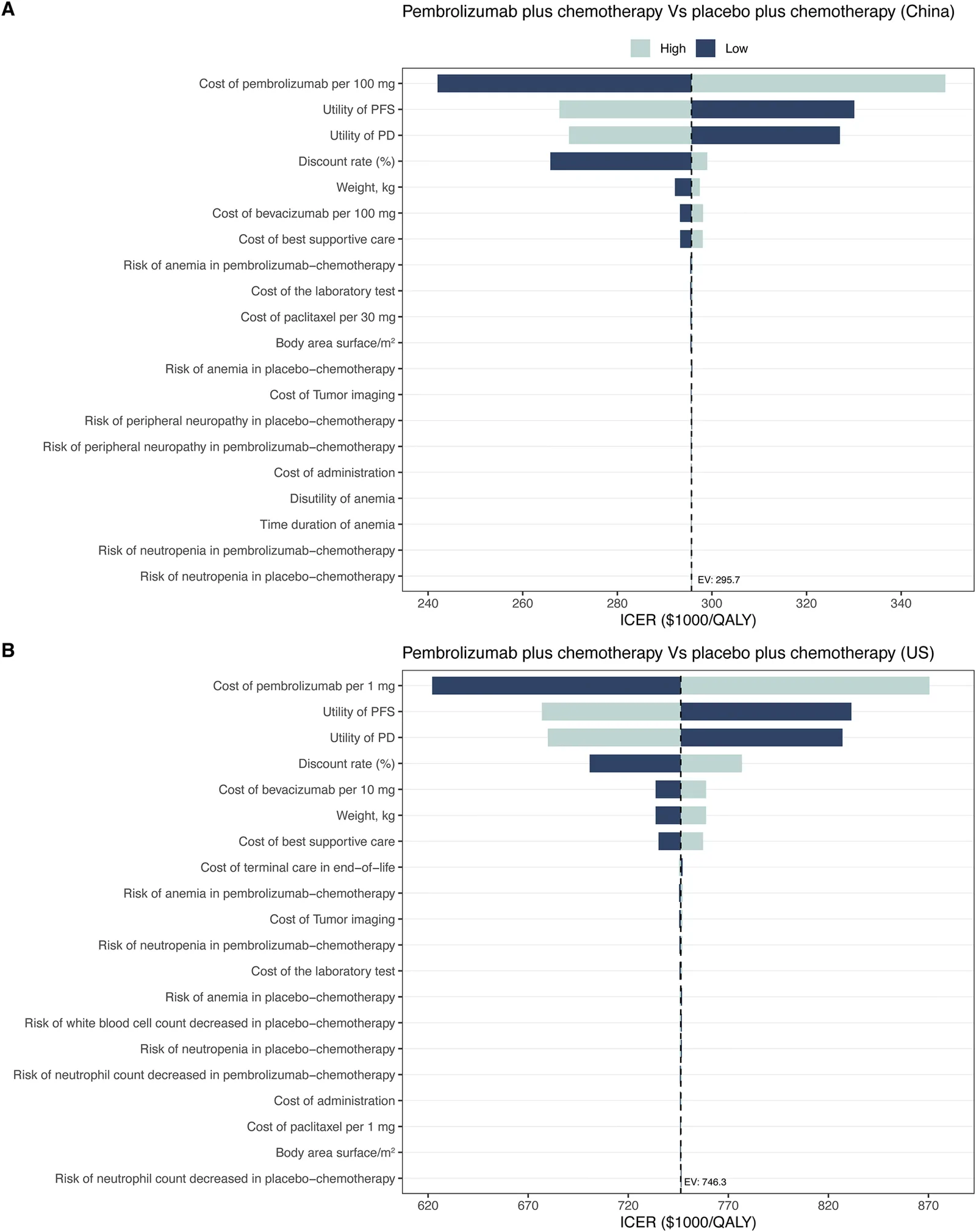
Tornado diagram for one-way sensitivity analysis in the PD-L1 CPS of one or higher population. **(A)** China. **(B)** The US. Abbreviation: PFS, progression-free survival; PD, progressed disease; ICER, incremental cost-effectiveness ratio; QALY, quality-adjusted life year; CPS, combined positive score.

#### 3.2.2 Two-way sensitivity analysis

The two-way deterministic analysis examined the joint effects of varying pembrolizumab and bevacizumab prices from zero to their respective base-case values while holding all other model parameters constant (Figure 3). In China, at the WTP threshold of $27,906/QALY, pembrolizumab plus weekly paclitaxel was cost-effective only within a narrow low-price region (Figure 3A). When bevacizumab was priced at its current level of $139.50 per 100 mg, the pembrolizumab price would need to decrease to no more than $5.27 per 100 mg. Even if bevacizumab were priced at $0, pembrolizumab would remain cost-effective only when priced below $119.14 per 100 mg. At the current pembrolizumab price of $2,507.87 per 100 mg, the regimen remained above the China-specific WTP threshold across all bevacizumab prices tested. In the US, a similar pattern was observed at the WTP threshold of $150,000/QALY (Figure 3B). When bevacizumab was priced at its current level of $73.20 per 10 mg, pembrolizumab would need to decrease to no more than $2.40 per mg to become cost-effective. If bevacizumab were priced at $0, the maximum cost-effective pembrolizumab price increased to $8.51 per mg. However, at the current pembrolizumab price of $60.29 per mg, the regimen exceeded the WTP threshold regardless of the bevacizumab price. In the overall-population scenario, the two-way deterministic analysis showed consistent findings (Supplementary Figure S4; Supplementary Figure S5). Overall, these results were consistent with the base-case and sensitivity analyses, suggesting that pembrolizumab plus weekly paclitaxel would require substantial price reductions to become cost-effective in either China or the US.

**FIGURE 3:**
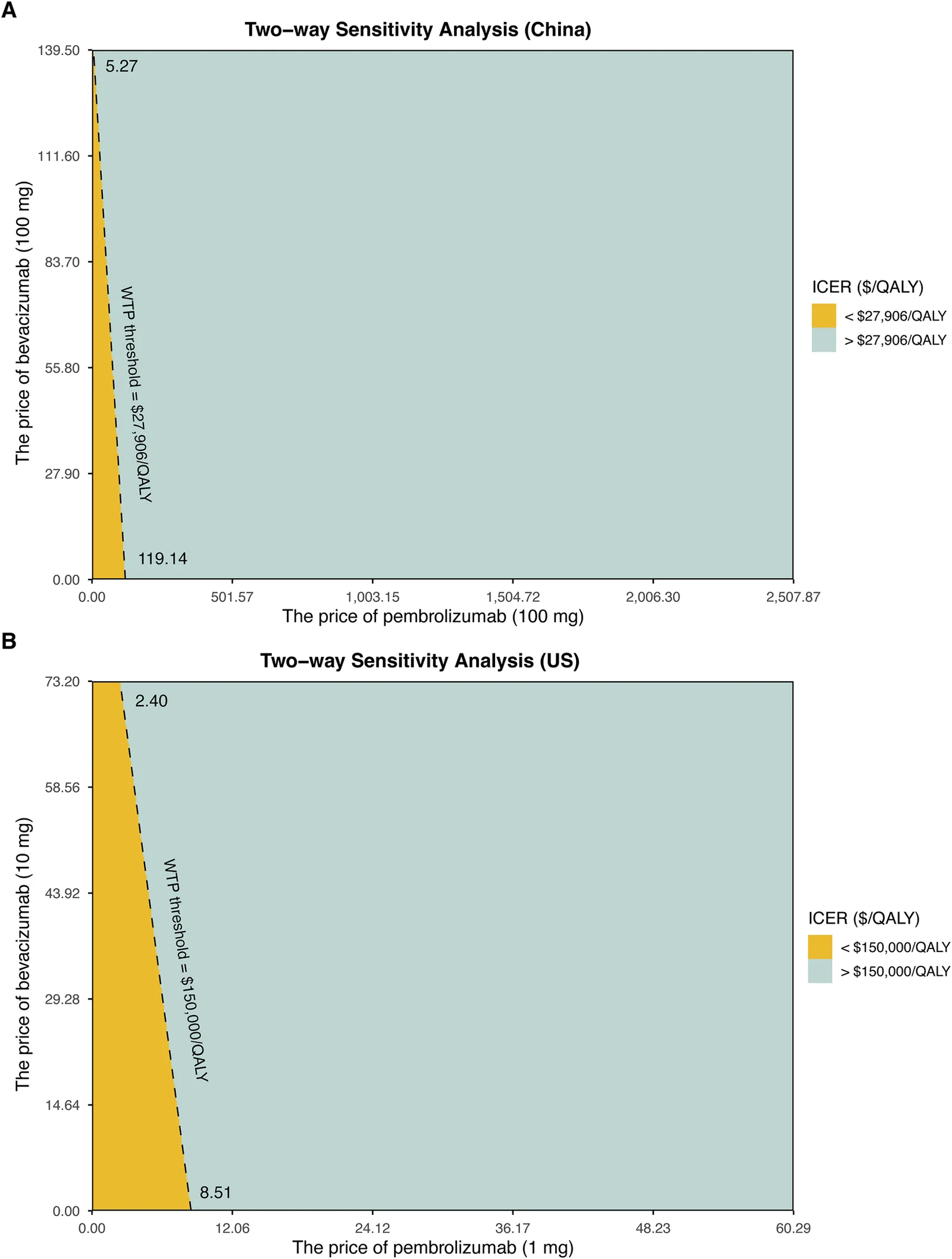
Two-way deterministic analysis in the PD-L1 CPS of one or higher population. **(A)** The two-way deterministic analysis of pembrolizumab and bevacizumab in China. **(B)** The two-way deterministic analysis of pembrolizumab and bevacizumab in the US. Abbreviation: QALY, quality-adjusted life year; WTP, willingness-to-pay.

#### 3.2.3 Probabilistic sensitivity analysis

Probabilistic sensitivity analysis using 1,000 Monte Carlo iterations is shown in Figure 4. In China, all simulated estimates for pembrolizumab plus weekly paclitaxel versus placebo plus chemotherapy were located in the northeast quadrant, indicating higher costs and greater QALYs (Figure 4A). Incremental QALYs were consistently positive, whereas incremental costs remained substantially above the monetary value of these health gains at the China-specific WTP threshold of $27,906/QALY. A similar pattern was observed in the US. Across 1,000 simulations, pembrolizumab plus weekly paclitaxel consistently generated additional QALYs but also higher costs than placebo plus chemotherapy. However, the incremental costs exceeded the health gains valued at the US WTP threshold of $150,000/QALY in all iterations (Figure 4B).

**FIGURE 4:**
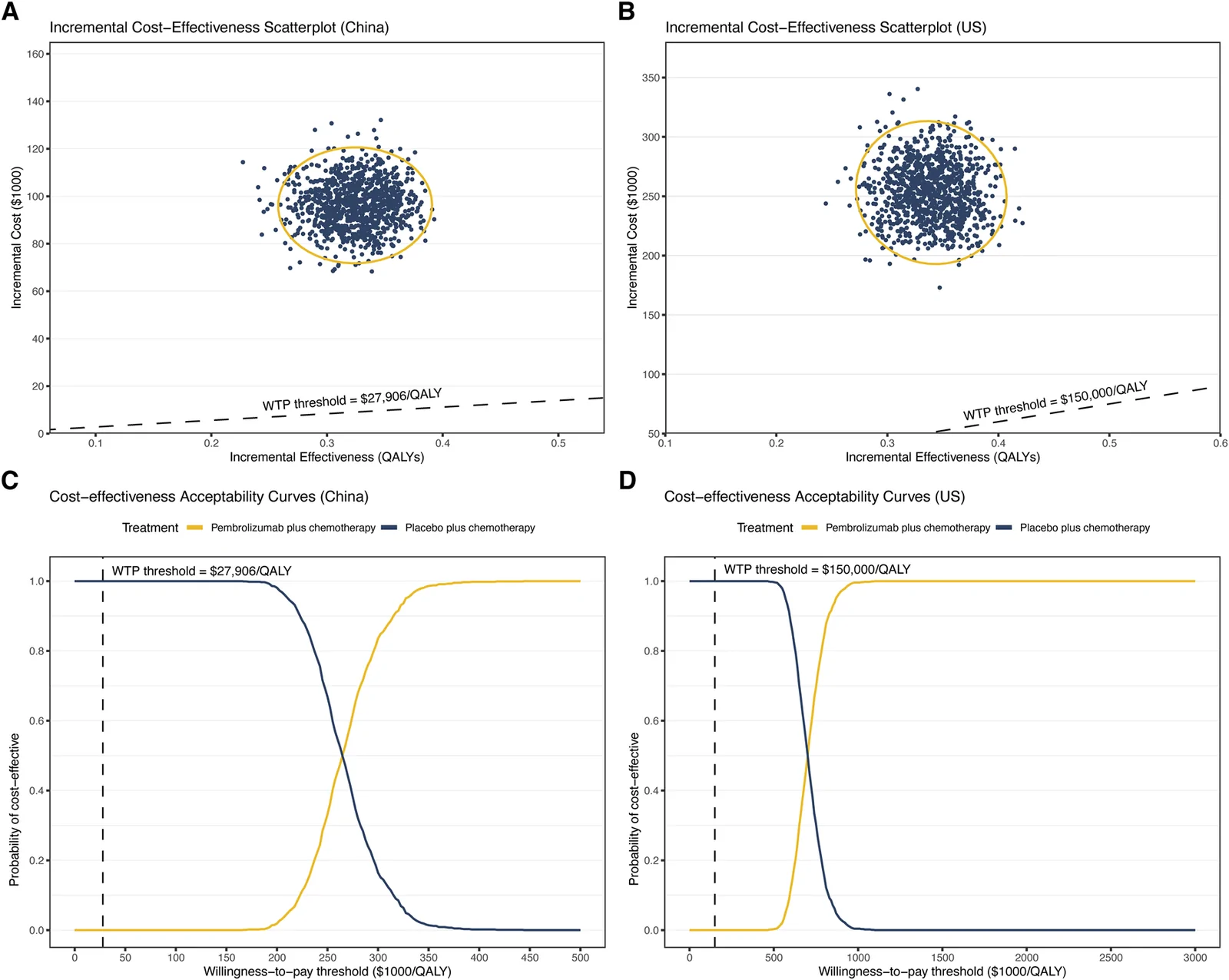
Probabilistic sensitivity analysis in the PD-L1 CPS of one or higher population. **(A)** The incremental cost-effectiveness scatter plot in China. **(B)** The incremental cost-effectiveness scatter plot in the US. **(C)** The cost-effectiveness acceptability curve in China. **(D)** The cost-effectiveness acceptability curve in the US. Abbreviation: QALY, quality-adjusted life year; WTP, willingness-to-pay.

The cost-effectiveness acceptability curves are shown in Figures 4C,D. In China, at the prespecified WTP threshold of $27,906/QALY, the probability that pembrolizumab plus chemotherapy was cost-effective was 0%, whereas placebo plus chemotherapy had a probability of 100%. The probability for pembrolizumab plus chemotherapy remained 0% until the WTP threshold exceeded $190,000/QALY, increased to 50.5% at $295,000/QALY, and reached 100% at $440,000/QALY. In the US, pembrolizumab plus chemotherapy also had a 0% probability of being cost-effective at the prespecified WTP threshold of $150,000/QALY, while placebo plus chemotherapy had a probability of 100%. The cost-effectiveness probability for pembrolizumab plus chemotherapy remained 0% through $480,000/QALY, increased to 53.1% at $750,000/QALY, and reached 100% at $1,160,000/QALY.

### 3.3 Scenario analyses

Scenario analyses were consistent with the base-case findings (Table 2). When the second-best-fitting parametric survival distributions were used, pembrolizumab plus weekly paclitaxel remained more costly and more effective than placebo plus chemotherapy in both countries. In China, the regimen increased costs from $31,249.20 to $127,168.27 and increased QALYs from 1.02 to 1.34, yielding an ICER of $295,769.00/QALY. This value exceeded the China-specific WTP threshold of $27,906/QALY. The INMB and INHB were −$86,869.04 and −3.11 QALYs, respectively. In the US, costs increased from $199,524.73 to $451,897.51, and QALYs increased from 1.03 to 1.37. The corresponding ICER was $746,444.87/QALY, exceeding the US WTP threshold of $150,000/QALY, with an INMB of −$201,657.82 and an INHB of −1.34 QALYs.

In the overall-population scenario, pembrolizumab plus weekly paclitaxel also failed to meet cost-effectiveness thresholds in both settings. In China, the regimen increased costs from $31,101.59 to $123,865.12 and QALYs from 1.01 to 1.29, yielding an ICER of $343,013.13/QALY. The INMB was −$85,216.70, and the INHB was −3.05 QALYs. In the US, costs increased from $198,745.55 to $440,023.69, and QALYs increased from 1.02 to 1.31. The ICER was $855,796.16/QALY, with an INMB of −$198,988.03 and an INHB of −1.33 QALYs. Sensitivity analyses supported these findings. In the one-way sensitivity analysis, ICERs remained above the WTP thresholds in China and the US across all tested parameter ranges (Supplementary Figure S6; Supplementary Figure S7). The cost of pembrolizumab was the most influential parameter, followed by the utilities of PFS and PD. In the probabilistic sensitivity analysis, most simulations were above the WTP threshold lines (Supplementary Figure S8), and the cost-effectiveness acceptability curves showed a near-zero probability that pembrolizumab plus weekly paclitaxel would be cost-effective at thresholds of $27,906/QALY in China and $150,000/QALY in the US (Supplementary Figure S9). These results were consistent with the base-case analysis in the PD-L1 CPS ≥1 population.

### 3.4 Subgroup analysis

Subgroup results were generally consistent with the base-case analysis (Table 3). Although more favorable ICERs were observed in several subgroups, including patients with ECOG performance status 1, previous PARP inhibitor use, and a platinum-free interval of 3–6 months, pembrolizumab plus weekly paclitaxel was not cost-effective in any subgroup in either country.

**TABLE 3:** Results of subgroup analyses.

Subgroup	OS HR (95% CI)	PFS HR (95% CI)	China				The United States			
			ICER ($/LY)	ICER ($/QALY)	INMB ($)	INHB (QALY)	ICER ($/LY)	ICER ($/QALY)	INMB ($)	INHB (QALY)
Age										
<65	0.72 (0.55, 0.93)	0.73 (0.55, 0.96)	146,590.82	234,985.78	−89,123.23	−3.19	375,619.71	604,158.60	−205,459.27	−1.37
≥65	0.85 (0.60, 1.11)	0.82 (0.58, 1.16)	260,980.87	407,926.83	−83,502.76	−2.99	638,749.31	1,002,732.49	−196,268.27	−1.31
ECOG performance status										
0	0.88 (0.66, 1.17)	0.83 (0.61, 1.12)	323,166.26	487,591.88	−82,940.83	−2.97	782,900.81	1,187,238.37	−195,864.36	−1.31
1	0.64 (0.47, 0.89)	0.72 (0.53, 0.99)	109,601.50	183,991.14	−89,709.88	−3.21	287,725.49	484,375.49	−202,885.59	−1.35
Previous PARP inhibitors										
Yes	0.70 (0.49, 0.99)	0.85 (0.59, 1.20)	123,852.47	224,231.30	−81,580.91	−2.92	315,708.64	572,704.12	−185,027.81	−1.23
No	0.79 (0.57, 1.10)	0.71 (0.54, 0.93)	202,901.51	294,503.02	−90,602.33	−3.25	511,647.16	745,994.71	−212,283.82	−1.42
PD−L1 CPS										
1 to <10	0.74 (0.56, 0.97)	0.79 (0.60, 1.04)	151,777.36	254,992.31	−85,212.03	−3.05	384,382.25	647,707.37	−196,263.96	−1.31
≥10	0.79 (0.57, 1.10)	0.72 (0.51, 1.01)	201,264.83	296,195.82	−89,897.42	−3.22	506,864.28	749,240.71	−210,458.19	−1.40
Bevacizumab use										
Yes	0.75 (0.58, 0.97)	0.75 (0.58, 0.97)	163,395.82	260,883.45	−87,806.04	−3.15	414,397.01	663,975.06	−203,401.05	−1.36
No	0.71 (0.49, 1.03)	0.75 (0.51, 1.11)	138,682.69	228,696.28	−87,756.31	−3.14	355,584.13	588,207.82	−201,440.06	−1.34
Platinum−free interval, months										
<3	0.85 (0.62, 1.15)	0.64 (0.47, 0.88)	298,569.38	344,327.53	−95,928.55	−3.44	749,214.79	870,023.21	−228,328.99	−1.52
3 to 6	0.71 (0.53, 0.96)	0.88 (0.65, 1.19)	126,015.58	232,629.25	−79,928.12	−2.86	319,530.04	590,830.89	−181,290.34	−1.21

Abbreviation: OS, overall survival; HR, hazard ratio; CI, confidence interval; PFS, progression-free survival; ICER, incremental cost-effectiveness ratio; LY, life-year; QALY, quality-adjusted life year; INMB, incremental net monetary benefits; INHB, incremental net health benefits; ECOG, Eastern Cooperative Oncology Group; CPS, combined positive score; PARP, poly ADP-ribose polymerase.

## 4 Discussion

In this cost-effectiveness analysis, pembrolizumab plus weekly paclitaxel improved survival and quality-adjusted survival but was unlikely to represent good economic value at current prices in either healthcare system. Ovarian cancer remains a major cause of cancer-related death among women worldwide. The recent burden estimates show that global incident cases increased from 0.1 million in 1990 to 0.2 million in 2021, while deaths increased by 84.5% over the same period (Yingying et al., 2025). Official Chinese health statistics released in 2025 reported national health expenditure of approximately 9.09 trillion yuan, or 6,454.4 yuan *per capita* and 6.7% of GDP (China Statistical Yearbook, 2025, 2025). In the US, ovarian cancer–related healthcare costs were estimated at $6.4 billion in 2020 (Cancer Trends Progress Report, 2025). One recent international economic analysis ranks China and the US first in absolute cancer-associated costs, at 24.1% and 20.8% of global totals, respectively (Simiao et al., 2023). For ovarian cancer specifically, global treatment expenditure is estimated at $51.9 billion over the 2022–2050 period (Simiao et al., 2023). Among the recent ovarian cancer economic evaluations we identified, including analyses of PARP inhibitors, mirvetuximab soravtansine, relacorilant, and first-line pembrolizumab-containing strategies, none evaluated pembrolizumab plus weekly paclitaxel for PD-L1–positive platinum-resistant recurrent ovarian cancer across both China and the US.(Kun et al., 2025; Shubin et al., 2026; Wolford et al., 2020; Youwen et al., 2024) In our base case, China gained 0.54 LYs and 0.32 QALYs with an ICER of $295,671.88/QALY, whereas the US gained 0.56 LYs and 0.34 QALYs with an ICER of $746,255.61/QALY. The corresponding INMB and INHB values were negative in China (−$86,873.48 and −3.11 QALYs) and in the US (−$201,670.44 and −1.34 QALYs). The regimen therefore delivers measurable health gains, but the incremental cost is disproportionate to the magnitude of benefit.

This finding should be interpreted against the backdrop of a disease setting with limited durable options and a high unmet need. Approximately 20%–30% of patients with ovarian cancer relapse or progress within 6 months of platinum-based chemotherapy (Lheureux et al., 2019). Among those with recurrent platinum-resistant disease, median OS is only 12–18 months, and conventional single-agent non-platinum chemotherapy yields response rates of roughly 10%–15% with a median PFS of about 3.5 months (Lheureux et al., 2019; St Laurent and Liu, 2024). Earlier PD-L1–based strategies in this space were largely disappointing. In AGO-OVAR 2.29/ENGOT-ov34, the addition of atezolizumab to bevacizumab and non-platinum chemotherapy did not significantly improve OS or PFS (Marmé et al., 2024). The JAVELIN Ovarian 200 also showed no significant survival advantage for avelumab with or without pegylated liposomal doxorubicin over chemotherapy alone, with median PFS of 3.7 vs. 3.5 months and median OS of 15.7 vs. 13.1 months for the combination and control groups (Pujade-Lauraine et al., 2021). By contrast, ENGOT-ov65/KEYNOTE-B96 established pembrolizumab plus paclitaxel, with or without bevacizumab, as a clinically important immunotherapy-based strategy for PD-L1–positive (CPS ≥1) disease, improving median PFS from 7.2 to 8.3 months and median OS from 14.0 to 18.2 months (Colombo et al., 2026). Despite its favorable efficacy, pembrolizumab plus paclitaxel presents a value challenge because its clinical gains are accompanied by substantially higher costs. We found that pembrolizumab plus weekly paclitaxel extended survival and quality-adjusted survival, but the incremental benefits did not offset the additional expenditure at current prices in either healthcare system. Thus, the regimen was unlikely to be cost-effective in both China and the US.

These findings are broadly concordant with prior economic evaluations of novel therapies in recurrent ovarian cancer. In a foundational recurrent-disease analysis, Wolford and colleagues found that non-platinum intravenous chemotherapy was the most cost-effective strategy at $6,412 per progression-free-survival month, compared with $12,187 for bevacizumab-containing regimens, $18,970 for niraparib, $16,327 for olaparib, and $16,637 for rucaparib, with PARP-inhibitor ICERs approximately 3- to 3.5-fold higher than those of intravenous non-platinum regimens (Wolford et al., 2020). A similar pattern was seen in a cost-effectiveness study of mirvetuximab soravtansine in FRα-high platinum-resistant recurrent ovarian cancer. Despite clear efficacy in a biomarker-selected population, ICERs were $596,189/QALY overall, $530,061/QALY in bevacizumab-naive disease, and $1,011,310/QALY in bevacizumab-pretreated disease (Youwen et al., 2024). The authors estimated that a price reduction of more than 75% would be needed for cost-effectiveness. Even relacorilant plus nab-paclitaxel, a regimen much closer to conventional US thresholds, still generated an ICER of $161,753/QALY for an incremental gain of only 0.27 QALYs, with drug price again emerging as the dominant determinant (Shubin et al., 2026). By contrast, first-line pembrolizumab-containing therapy in BRCA wild-type, LOH-low advanced ovarian cancer appeared more favorable in selected settings, with ICERs of $568,586/QALY overall and $399,919/QALY in PD-L1 CPS–high disease in the US, but $34,032/QALY overall and $18,462/QALY in PD-L1 CPS–high disease in China (Kun et al., 2025). Taken together, these comparisons suggest that disease setting, biomarker enrichment, and survival horizon might shape value, and that in platinum-resistant recurrent disease the combination of shorter survival and smaller absolute QALY gains might make cost-effectiveness particularly difficult to achieve.

The deterministic sensitivity analyses clarify why the base-case ICERs remained unfavorable. Pembrolizumab cost, PFS utility, and PD utility were the most influential parameters in both countries. Mechanistically, pembrolizumab price dominates because repeated dosing of an expensive immune checkpoint inhibitor rapidly accumulates cost, and longer time spent progression-free can paradoxically extend the duration over which high-priced therapy is delivered. The utility inputs were also central because when incremental QALY gains are modest, even plausible variation in progression-free and progressed-disease quality-of-life weights materially changes the ICER denominator. This pattern is consistent with newer ovarian cancer CEAs where drug acquisition cost and health-state utilities repeatedly emerge as the dominant ICER drivers (Kun et al., 2025; Shubin et al., 2026; Yamin et al., 2022). The probabilistic and scenario analyses further indicate that the unfavorable conclusion was robust rather than model-fragile. At the prespecified WTP thresholds, the probability of cost-effectiveness was zero in both settings. Cost-effectiveness was not achieved until thresholds exceeded approximately $190,000 per QALY in China and $480,000 per QALY in the US. Alternative parametric survival assumptions and extension to the overall trial population did not change the economic conclusion. No subgroup in either country achieved cost-effectiveness, and all INMB and INHB values remained negative. Lower ICERs were nevertheless found in patients with an ECOG performance status of 1, in those with previous PARP inhibitor use, and in those whose platinum-free interval was 3–6 months. However, these subgroup signals should be treated cautiously. Subgroup HRs are exploratory rather than reimbursement-defining. Future economic stratification will probably require composite selection incorporating PD-L1, resistance pattern, prior PARP exposure, and other tumor or immune characteristics rather than PD-L1 alone.

Among all scenario analyses, the two-way deterministic analysis was particularly relevant to pricing and reimbursement decisions. At current bevacizumab prices, pembrolizumab would need to fall to $5.27 per 100 mg in China and to $2.40 per mg in the US to meet the corresponding WTP thresholds. Even if bevacizumab were priced at zero, pembrolizumab would still need to fall below $119.14 per 100 mg in China and below $8.51 per mg in the US, indicating that the principal barrier to value is pembrolizumab pricing rather than ancillary monitoring, intravenous drug administration, or AE costs. In neutral policy terms, these findings support discussion of value-based pricing, national price negotiations, indication-specific discounts, and patient assistance program, but they do not justify blanket claims that the regimen should or should not be used in all individual patients. Overall, the two-way deterministic analysis suggests that substantial price reductions for pembrolizumab and bevacizumab would be required before a value-based price could be justified.

Against the backdrop of the ENGOT-ov65/KEYNOTE-B96 trial and its subsequent clinical implementation, this study provides a timely, decision-relevant cross-national economic evaluation of pembrolizumab plus weekly paclitaxel for PD-L1-positive platinum-resistant recurrent ovarian cancer. Nonetheless, these findings should be interpreted in light of several limitations. First, survival beyond observed follow-up required parametric extrapolation. Although the alternative extrapolation scenario was reassuringly consistent, structural uncertainty cannot be fully eliminated. Second, because utility estimates were derived from external literature rather than collected within the trial, their influence on the model may not fully reflect health-related quality of life specific to PD-L1–positive platinum-resistant recurrent ovarian cancer. Third, cost inputs, reimbursement rules, and procurement prices change over time, so the economic conclusion is current-price specific rather than permanent. Fourth, post-progression management was simplified because the published ENGOT-ov65/KEYNOTE-B96 data did not provide sufficiently detailed, treatment-arm-specific information on subsequent anticancer regimens, treatment proportions, dosing schedules, treatment durations, or country-specific resource use. Best supportive care costs were accrued during each cycle spent in the PD state, whereas terminal-care costs were applied once at death. This approach may not fully capture the costs of active subsequent anticancer treatment or potential differences in post-progression management between the treatment groups. Fifth, AE modelling was simplified by including only grade 3 or higher events with an incidence of at least 5% in either treatment group and by applying their costs and disutilities as one-time events during the first model cycle. Although this approach captured the most common severe toxicities reported in ENGOT-ov65/KEYNOTE-B96, it may have excluded rare, delayed, recurrent, or prolonged AEs, particularly immune-related toxicities associated with pembrolizumab that may require extended monitoring, corticosteroid treatment, hospitalization, or specialist care. Finally, the model was designed for the US and Chinese healthcare systems and should not be generalized automatically to differently financed settings. Even so, because the base-case ICERs were so far above the prespecified thresholds, modest variation in these assumptions is unlikely to reverse the principal economic conclusion.

## 5 Conclusion

In summary, compared with placebo plus weekly paclitaxel, pembrolizumab plus weekly paclitaxel provided clinically meaningful gains in survival and quality-adjusted survival for patients with PD-L1–positive platinum-resistant recurrent ovarian cancer. However, under the current drug prices, model assumptions, and country-specific WTP thresholds, the pembrolizumab-containing strategy was unlikely to be cost-effective in either China or the US. Substantial reductions in the acquisition prices of pembrolizumab and bevacizumab would be required for this strategy to become cost-effective relative to placebo plus weekly paclitaxel.

## Data Availability

The original contributions presented in the study are included in the article/Supplementary Material, further inquiries can be directed to the corresponding authors.
