# Supplementary material for: Cost-effectiveness of pembrolizumab plus weekly paclitaxel for PD-L1–positive platinum-resistant recurrent ovarian cancer in the United States and China
Source: Front Pharmacol. 2026 Sep 21;17:1926275. doi: 10.3389/fphar.2026.1926275 (PMC13635326; doi:10.3389/fphar.2026.1926275)
Supplement: Supplementary file 1 [file DataSheet1.pdf]

Supplementary File:

Cost-effectiveness of pembrolizumab plus weekly paclitaxel for PD-L1–positive platinum-resistant recurrent ovarian cancer in the United States and China

Table S1. CHEERS 2022 Checklist.[1] 2
Table S2. Parametric survival distributions evaluated for progression-free survival and overall survival in the PD-L1 CPS of 1 or higher population. 5
Table S3. Duration parameters for adverse events included in the model. 6
Figure S1. The reconstructed Kaplan-Meier curves for overall (A) and progression free (B) survival for PD-L1–positive platinum-resistant recurrent ovarian cancer. 7
Figure S2. Reconstructed Kaplan–Meier estimates and fitted parametric survival curves of overall (A) and progression free (B) survival for pembrolizumab-chemotherapy group in PD-L1–positive platinum-resistant recurrent ovarian cancer. 8
Figure S3. Reconstructed Kaplan–Meier estimates and fitted parametric survival curves of overall (A) and progression free (B) survival for placebo-chemotherapy group in PD-L1–positive platinum-resistant recurrent ovarian cancer. 9
Figure S4. The two-way deterministic analysis of pembrolizumab and bevacizumab in China among the overall population. 10
Figure S5. The two-way deterministic analysis of pembrolizumab and bevacizumab in US among the overall population. 11
Figure S6. The tornado diagram of one-way sensitivity analysis in China among the overall population. 12
Figure S7. The tornado diagram of one-way sensitivity analysis in US among the overall population. 13
Figure S8. The cost-effectiveness probabilistic scatter plot in China (A) and the US (B) among the overall population. 14
Figure S9. The cost-effectiveness acceptability curve in China (A) and the US (B) among the overall population. 15
Reference 16

Table S1. CHEERS 2022 Checklist.[1]

Topic	No.	Item	Reported
**Title**	1	Identify the study as an economic evaluation and specify the interventions being compared.	Yes
**Abstract**	2	Provide a structured summary that highlights context, key methods, results, and alternative analyses.	Yes
**Introduction**			
**Background and objectives**	3	Give the context for the study, the study question, and its practical relevance for decision making in policy or practice.	Yes
**Methods**			
**Health economic analysis plan**	4	Indicate whether a health economic analysis plan was developed and where available.	Not applicable
**Study population**	5	Describe characteristics of the study population (such as age range, demographics, socioeconomic, or clinical characteristics).	Yes
**Setting and location**	6	Provide relevant contextual information that may influence findings.	Yes
**Comparators**	7	Describe the interventions or strategies being compared and why chosen.	Yes
**Perspective**	8	State the perspective(s) adopted by the study and why chosen.	Yes
**Time horizon**	9	State the time horizon for the study and why appropriate.	Yes
**Discount rate**	10	Report the discount rate(s) and reason chosen.	Yes
**Selection of outcomes**	11	Describe what outcomes were used as the measure(s) of benefit(s) and harm(s).	Yes
**Measurement of outcomes**	12	Describe how outcomes used to capture benefit(s) and harm(s) were measured.	Yes
**Valuation of outcomes**	13	Describe the population and methods used to measure and value outcomes.	Yes
**Measurement and valuation of resources and costs**	14	Describe how costs were valued.	Yes
**Currency, price date, and conversion**	15	Report the dates of the estimated resource quantities and unit costs, plus the currency and year of conversion.	Yes

Topic	No.	Item	Reported
**Rationale and description of model**	16	If modelling is used, describe in detail and why used. Report if the model is publicly available and where it can be accessed.	Yes
**Analytics and assumptions**	17	Describe any methods for analysing or statistically transforming data, any extrapolation methods, and approaches for validating any model used.	Yes
**Characterising heterogeneity**	18	Describe any methods used for estimating how the results of the study vary for subgroups.	Yes
**Characterising distributional effects**	19	Describe how impacts are distributed across different individuals or adjustments made to reflect priority populations.	Not applicable
**Characterising uncertainty**	20	Describe methods to characterise any sources of uncertainty in the analysis.	Yes
**Approach to engagement with patients and others affected by the study**	21	Describe any approaches to engage patients or service recipients, the general public, communities, or stakeholders (such as clinicians or payers) in the design of the study.	Not applicable
**Results**			
**Study parameters**	22	Report all analytic inputs (such as values, ranges, references) including uncertainty or distributional assumptions.	Yes
**Summary of main results**	23	Report the mean values for the main categories of costs and outcomes of interest and summarise them in the most appropriate overall measure.	Yes
**Effect of uncertainty**	24	Describe how uncertainty about analytic judgments, inputs, or projections affect findings. Report the effect of choice of discount rate and time horizon, if applicable.	Yes
**Effect of engagement with patients and others affected by the study**	25	Report on any difference patient/service recipient, general public, community, or stakeholder involvement made to the approach or findings of the study	Not applicable
**Discussion**			
**Study findings, limitations, generalisability, and current knowledge**	26	Report key findings, limitations, ethical or equity considerations not captured, and how these could affect patients, policy, or practice.	Yes
**Other relevant information**			

Topic	No.	Item	Reported
**Source of funding**	27	Describe how the study was funded and any role of the funder in the identification, design, conduct, and reporting of the analysis	Yes
**Conflicts of interest**	28	Report authors conflicts of interest according to journal or International Committee of Medical Journal Editors requirements.	Yes

Table S2. Parametric survival distributions evaluated for progression-free survival and overall survival in the PD-L1 CPS of 1 or higher population.

Parameters	Exponential	Weibull	Gamma	Log-logistic	Log-normal	Gompertz	Gen gamma
**OS in pembrolizumab-chemotherapy group**							
AIC	1460.68	1435.13	1430.667	1426.586	1428.761	1450.656	1429.199
BIC	1464.135	1442.041	1437.578	1433.497	1435.672	1457.567	1439.565
**PFS in pembrolizumab-chemotherapy group**							
AIC	1394.175	1382.817	1375.052	1355.019	1360.845	1395.762	1362.499
BIC	1397.63	1389.728	1381.963	1361.93	1367.755	1402.672	1372.865
**OS in placebo-chemotherapy group**							
AIC	1535.621	1507.149	1501.935	1496.685	1504.269	1524.751	1501.319
BIC	1539.068	1514.043	1508.829	1503.579	1511.163	1531.645	1511.659
**PFS in placebo-chemotherapy group**							
AIC	1352.39	1334.006	1323.613	1306.662	1308.154	1352.563	1310.013
BIC	1355.836	1340.9	1330.506	1313.555	1315.048	1359.457	1320.353

Abbreviation: AIC = Akaike information criterion. BIC = Bayesian information criterion. PFS = progression-free survival. OS = overall survival.

Table S3. Duration parameters for adverse events included in the model.

Adverse event	Duration, days	Range, days	Distribution	Basis/source
Anemia	51.21	40.97–61.46	Normal	Published economic evaluation[2, 3]
Peripheral neuropathy	21	16.8–25.2	Normal	Model assumption based on one paclitaxel treatment cycle
Fatigue	21	16.8–25.2	Normal	Model assumption based on one paclitaxel treatment cycle
White blood cell count decreased	6.35	5.08–7.62	Normal	Published economic evaluation[2, 3]
Neutropenia	6.35	5.08–7.62	Normal	Published economic evaluation[2, 3]
Neutrophil count decreased	6.35	5.08–7.62	Normal	Published economic evaluation[2, 3]

Note: ENGOT-ov65/KEYNOTE-B96 did not report event-specific AE durations. Therefore, duration estimates were obtained from published clinical or economic studies where available. The durations of peripheral neuropathy and fatigue were assumed to be 21 days, corresponding to one paclitaxel treatment cycle. Wider uncertainty ranges were used in the one-way sensitivity analysis to reflect the possibility of prolonged toxicity, particularly peripheral neuropathy.

Figure S1. The reconstructed Kaplan-Meier curves for overall (A) and progression free (B) survival for PD-L1–positive platinum-resistant recurrent ovarian cancer.

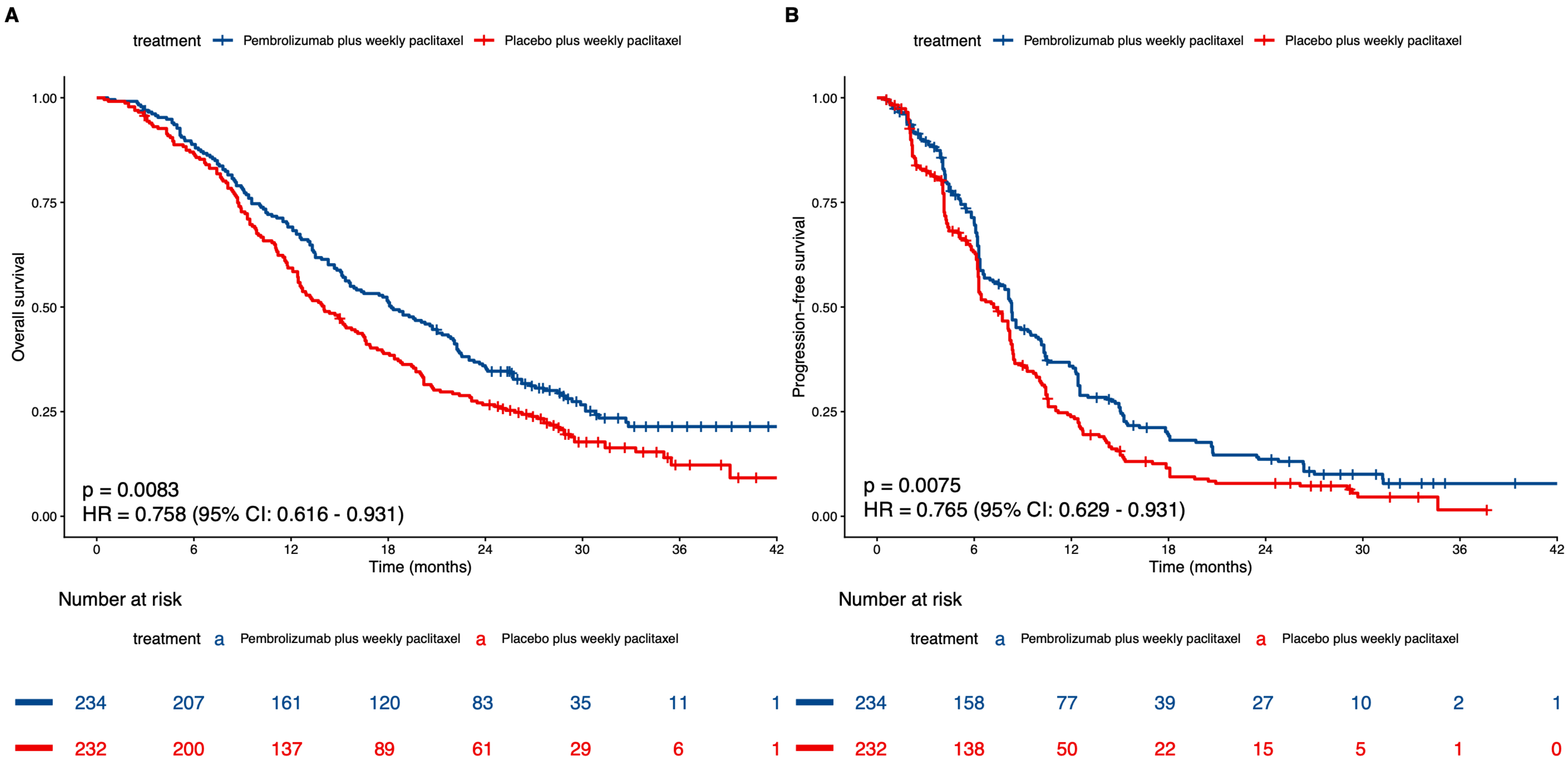

Note: The reconstructed Kaplan–Meier curves were visually compared with the published final-analysis curves from ENGOT-ov65/KEYNOTE-B96. Overall survival data for the PD-L1 combined positive score ≥1 population were obtained from Supplementary Figure S3 of the trial publication, and progression-free survival data were obtained from Supplementary Figure S4A.

Figure S2. Reconstructed Kaplan–Meier estimates and fitted parametric survival curves of overall (A) and progression free (B) survival for pembrolizumab-chemotherapy group in PD-L1–positive platinum-resistant recurrent ovarian cancer.

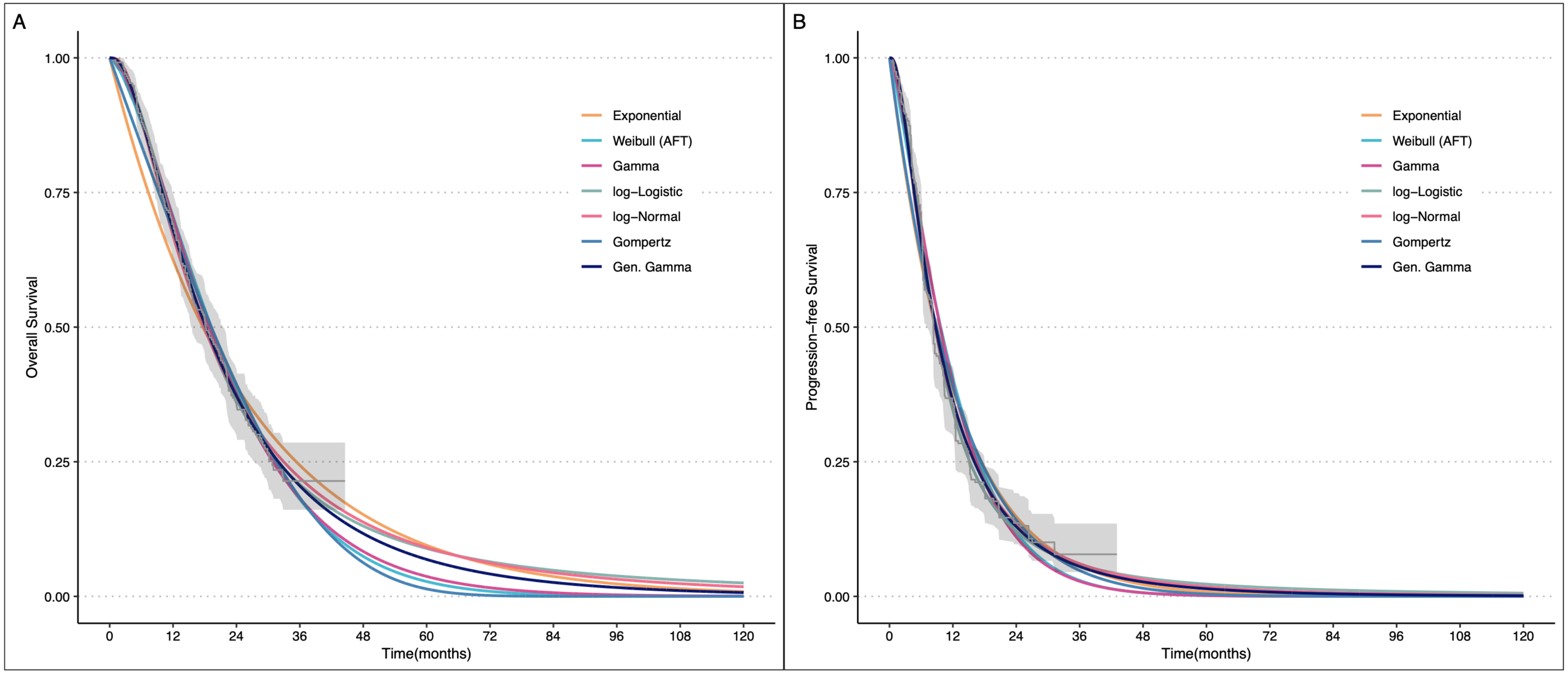

Figure S3. Reconstructed Kaplan–Meier estimates and fitted parametric survival curves of overall (A) and progression free (B) survival for placebo-chemotherapy group in PD-L1–positive platinum-resistant recurrent ovarian cancer.

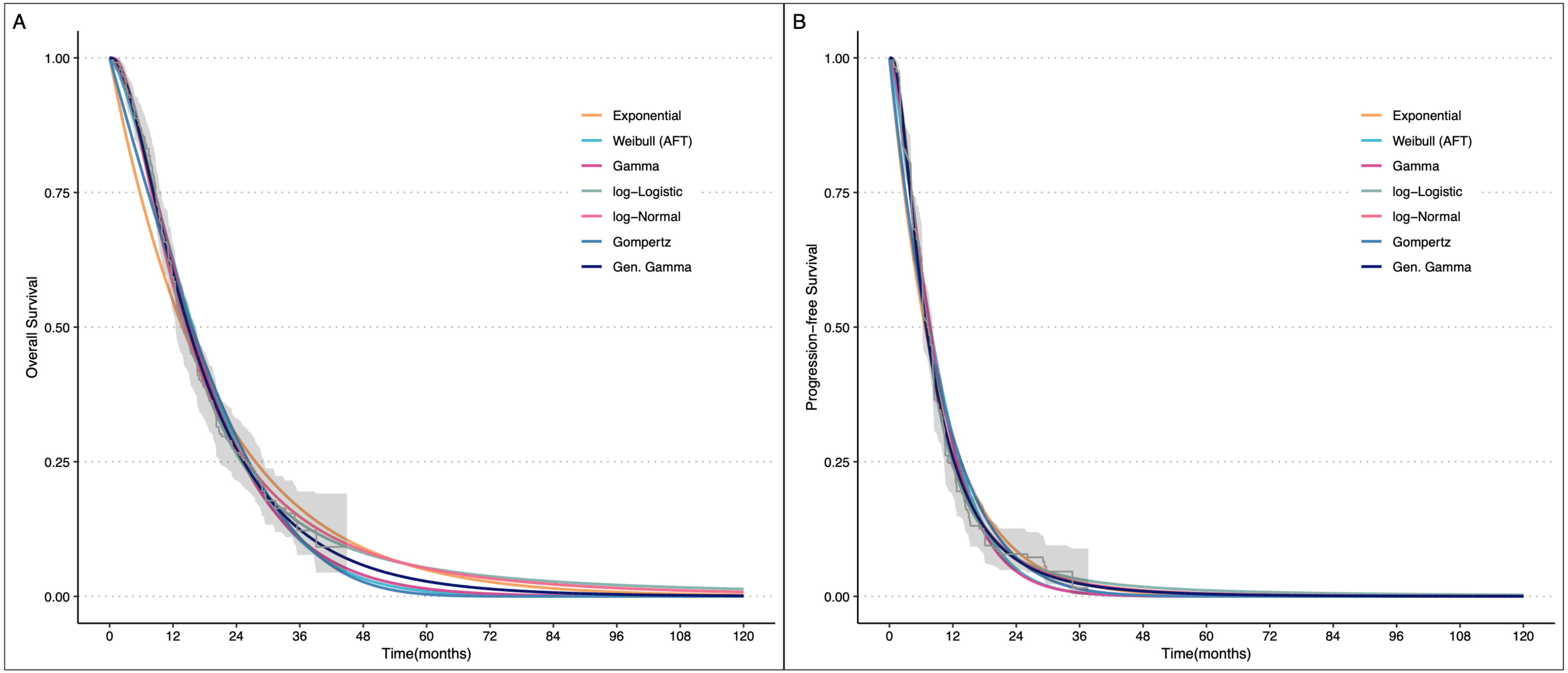

Figure S4. The two-way deterministic analysis of pembrolizumab and bevacizumab in China among the overall population**.**

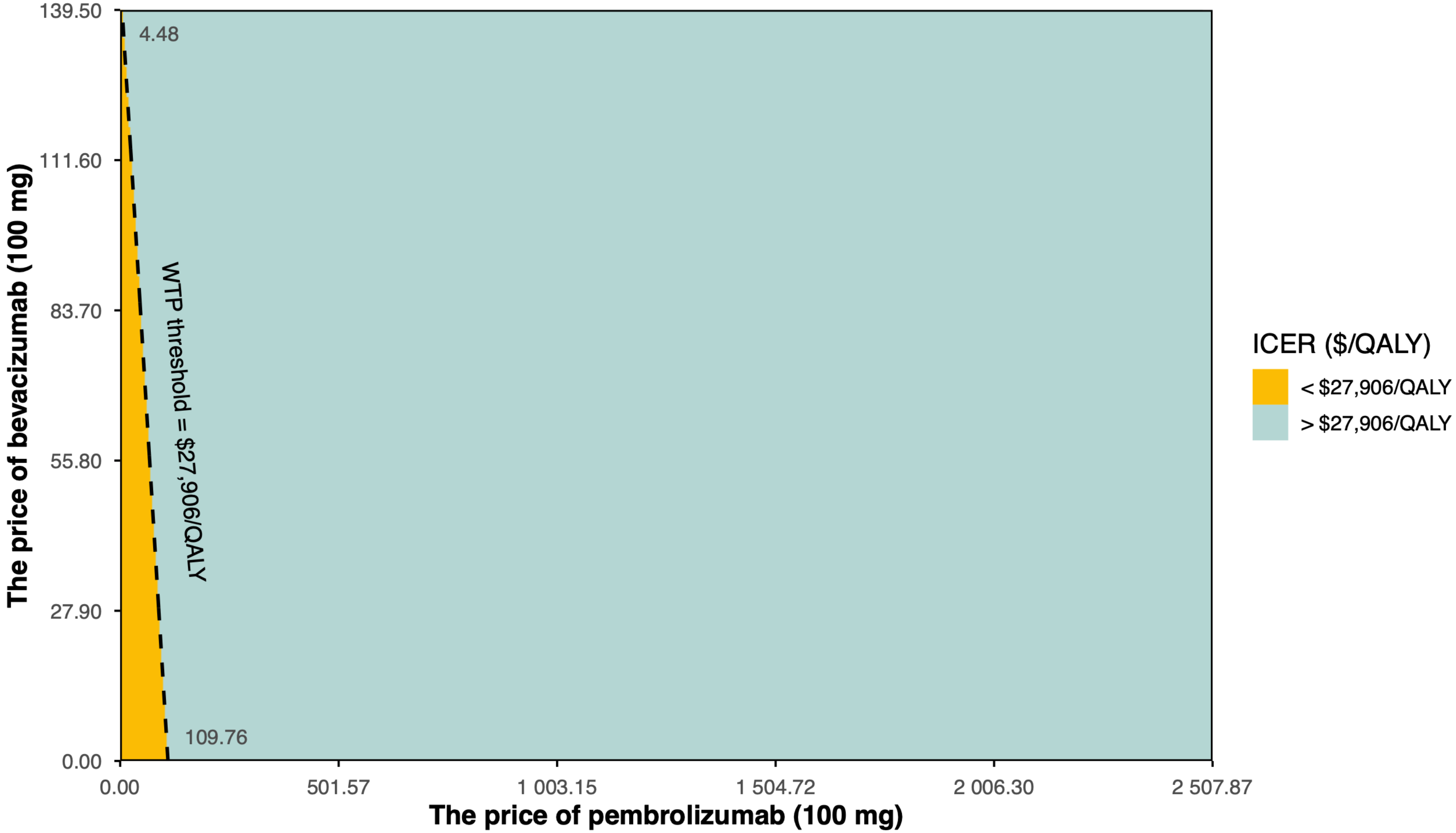

Figure S5. The two-way deterministic analysis of pembrolizumab and bevacizumab in US among the overall population.

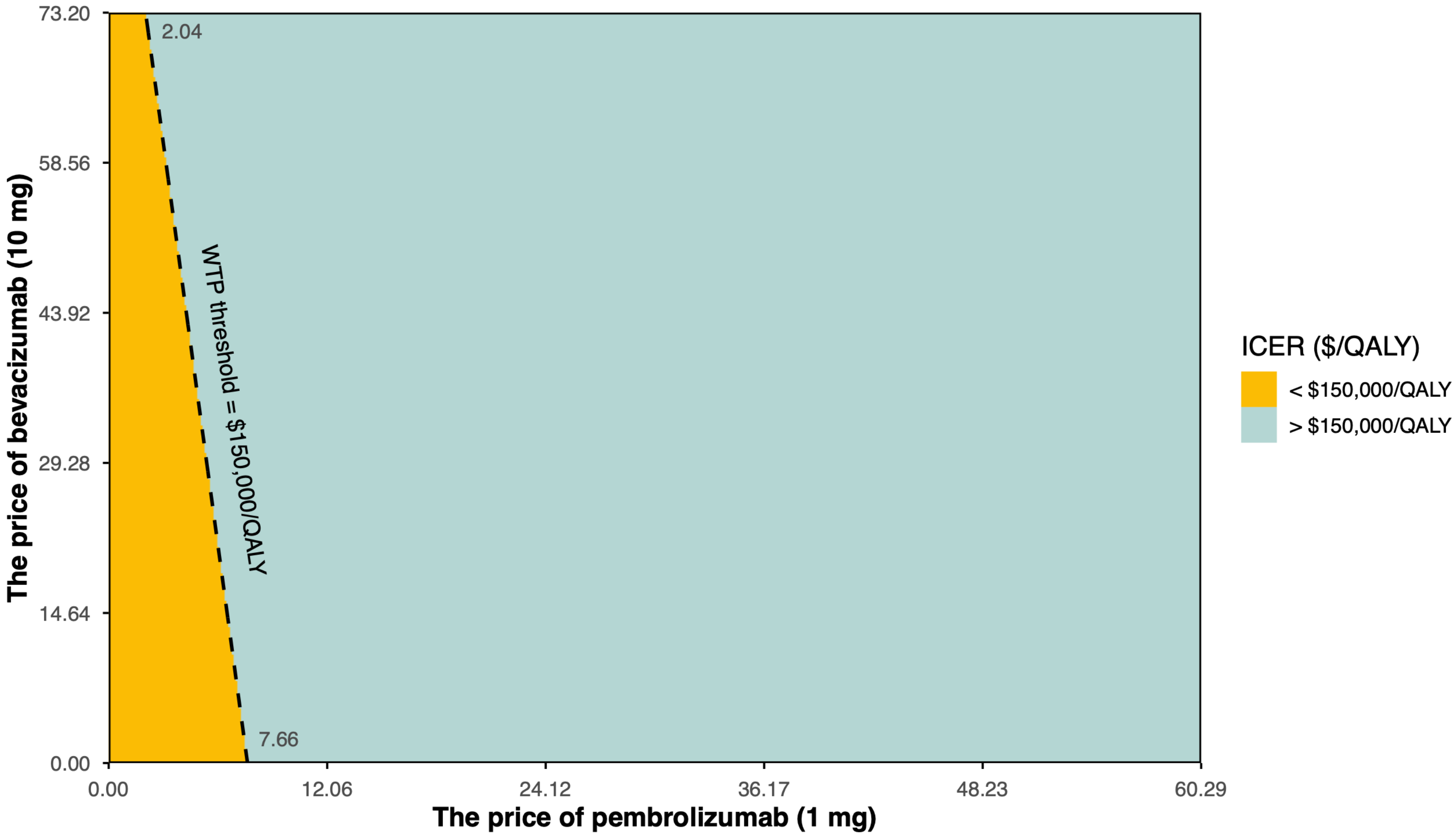

Figure S6. The tornado diagram of one-way sensitivity analysis in China among the overall population.

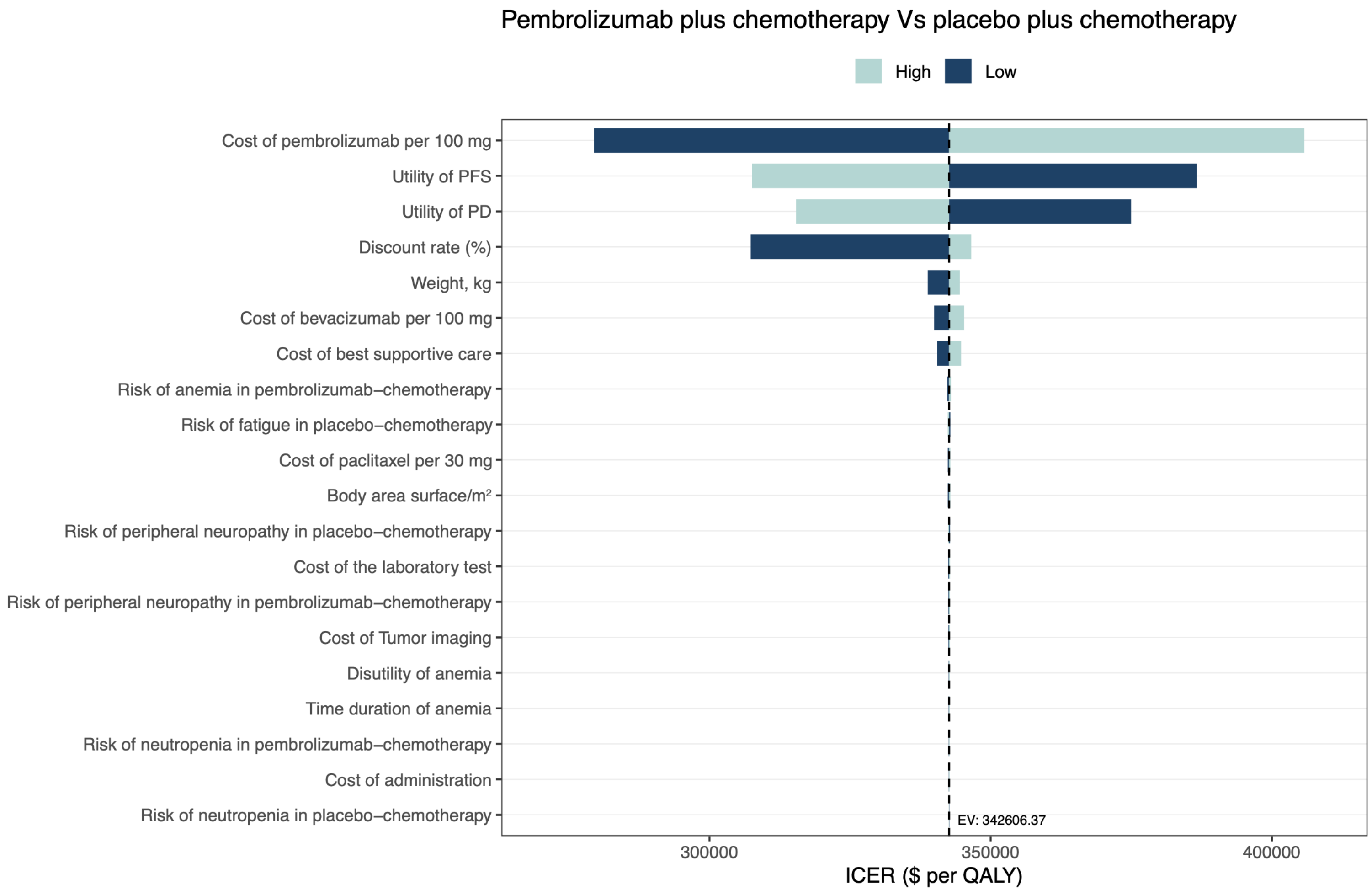

Figure S7. The tornado diagram of one-way sensitivity analysis in US among the overall population.

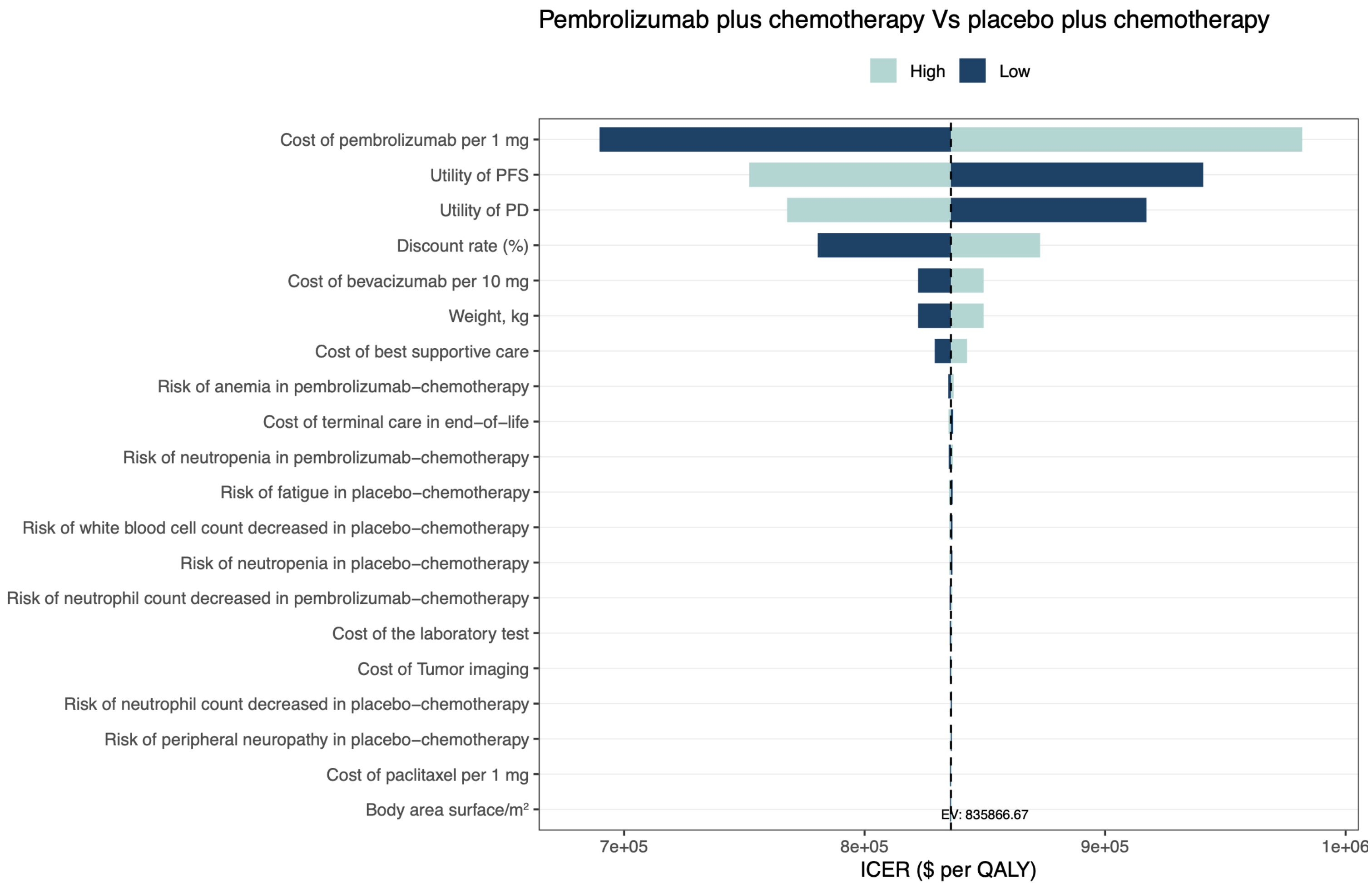

Figure S8. The cost-effectiveness probabilistic scatter plot in China (A) and the US (B) among the overall population.

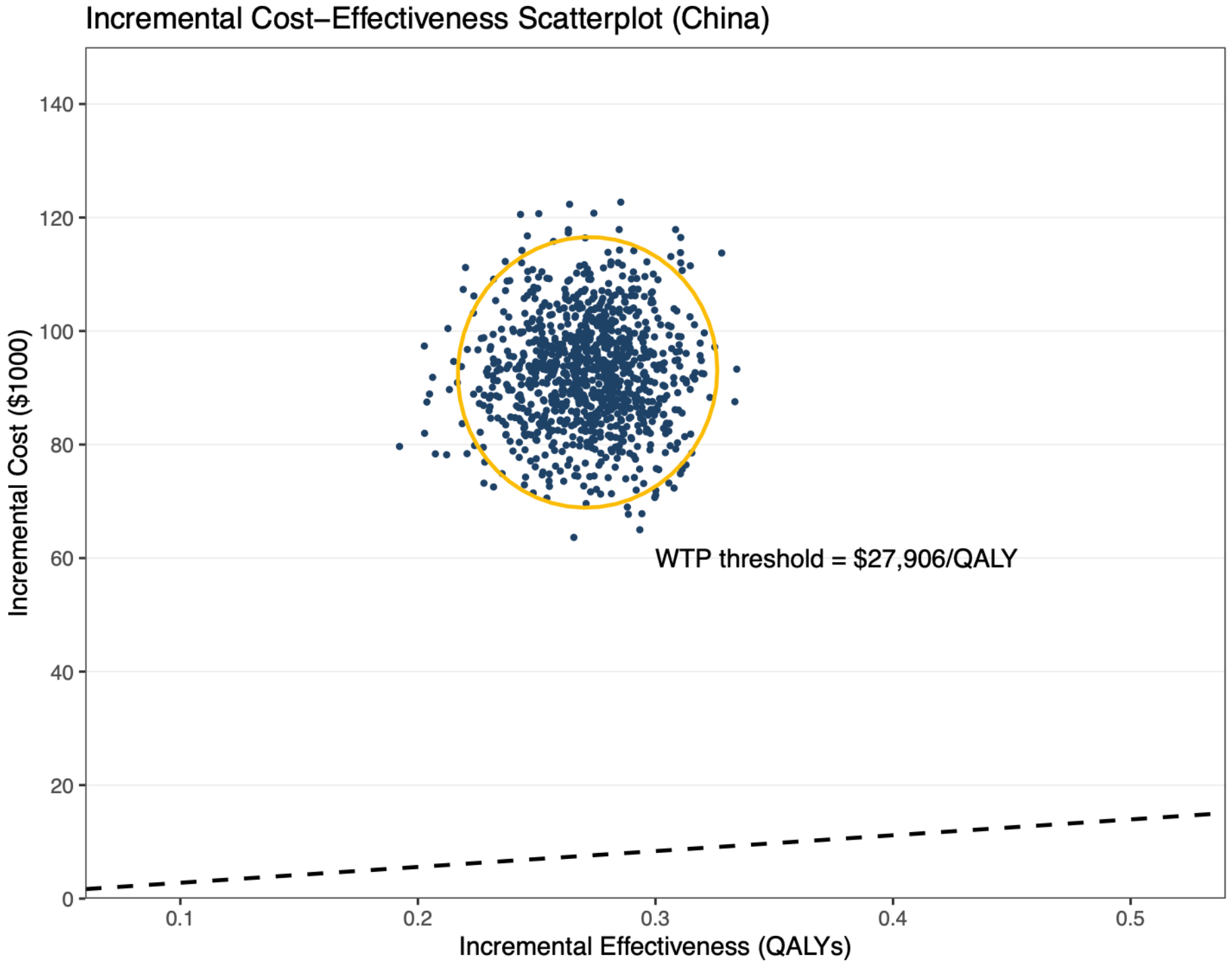

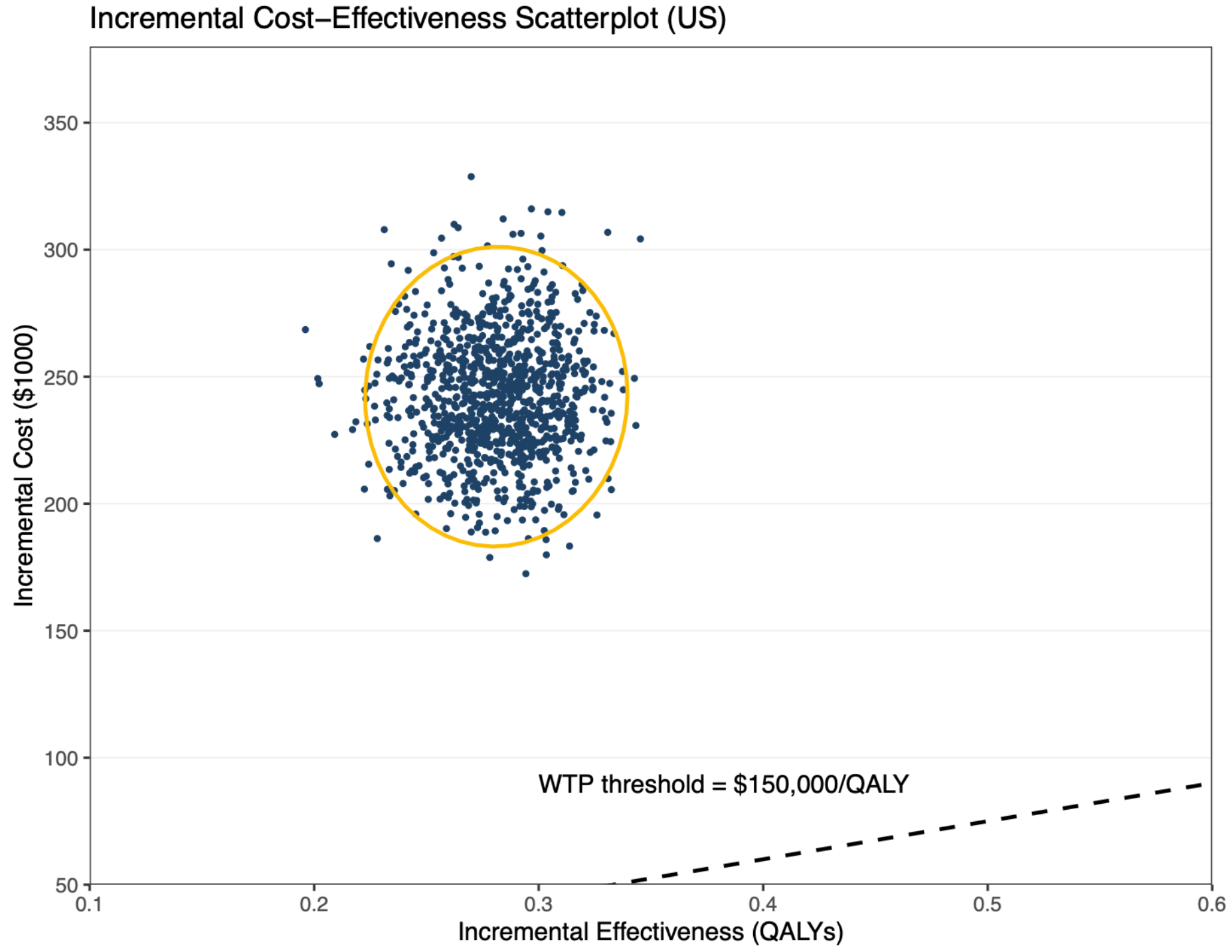

Figure S9. The cost-effectiveness acceptability curve in China (A) and the US (B) among the overall population.

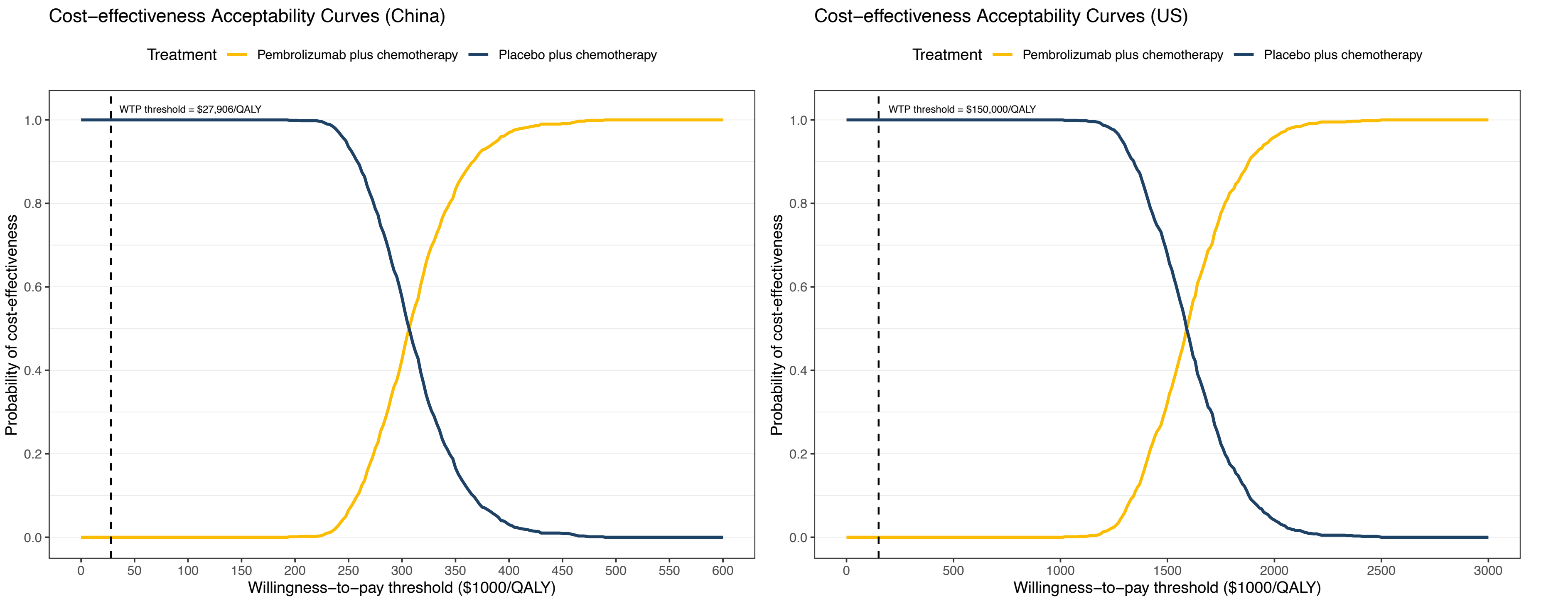

Reference

1. Husereau D, Drummond M, Augustovski F, de Bekker-Grob E, Briggs AH, Carswell C, et al. Consolidated Health Economic Evaluation Reporting Standards 2022 (CHEERS 2022) statement: updated reporting guidance for health economic evaluations. BMC Med. 2022;20(1):23. https://doi.org/10.1186/s12916-021-02204-0 PMID: 35022047
2. Zhao M, Shao T, Chi Z, Tang W. Effectiveness and cost-effectiveness analysis of 11 treatment paths, seven first-line and three second-line treatments for Chinese patients with advanced wild-type squamous non-small cell lung cancer: A sequential model. Front Public Health. 2023;11:1051484. https://doi.org/10.3389/fpubh.2023.1051484 PMID: 36908446
3. Rui M, Fei Z, Wang Y, Zhang X, Ma A, Sun H, et al. Cost-effectiveness analysis of sintilimab + chemotherapy versus camrelizumab + chemotherapy for the treatment of first-line locally advanced or metastatic nonsquamous NSCLC in China. J Med Econ. 2022;25(1):618-29. https://doi.org/10.1080/13696998.2022.2071066 PMID: 35475459
